# Synthesis, Hydration, Antibacterial, Antifungal, and Antiviral Properties of New Surface-Active Nicotine-Based Ionic Liquids

**DOI:** 10.3390/molecules31214001

**Published:** 2026-11-06

**Authors:** Andrija Vukov, Milana Rakić, Maja Karaman, Bartosz Kamiński, Jan Paczesny, Janez Cerar, Milan Vraneš

**Affiliations:** 1 Department of Chemistry, Biochemistry and Environmental Protection, Faculty of Sciences, University of Novi Sad, Trg Dositeja Obradovića 3, 21000 Novi Sad, Serbia; andrija.vukov@dh.uns.ac.rs; 2 Department of Biology and Ecology, Faculty of Sciences, University of Novi Sad, Trg Dositeja Obradovića 3, 21000 Novi Sad, Serbia; milana.novakovic@dbe.uns.ac.rs (M.R.); maja.karaman@dbe.uns.ac.rs (M.K.); 3 Institute of Physical Chemistry, Polish Academy of Sciences, Marcina Kasprzaka 44/52, 01-224 Warsaw, Poland; bkaminski@ichf.edu.pl (B.K.); jpaczesny@ichf.edu.pl (J.P.); 4 Faculty of Chemistry and Chemical Technology, University of Ljubljana, Večna pot 113, 1000 Ljubljana, Slovenia; janez.cerar@fkkt.uni-lj.si

**Keywords:** water, antimicrobial, density, viscosity, acoustic, surfactants

## Abstract

Nicotine is a pyridine alkaloid naturally produced by plants of the *Solanaceae* family. In this study, four nicotine-based ionic liquids (ILs) with different alkyl side-chain lengths were synthesized by alkylation with alkyl bromides: *N*-octylnicotinium bromide, *N*-decylnicotinium bromide, *N*-dodecylnicotinium bromide, and *N*-tetradecylnicotinium bromide; of these, only the octyl derivative was previously reported. Their structures were confirmed by NMR spectroscopy. All ILs were water-soluble within the investigated concentration range and exhibited surfactant properties. Critical micelle concentrations (CMC) were determined by conductometry, while density, sound velocity, and viscosity measurements were used to investigate hydration behavior. CMC values decreased with increasing alkyl chain length, indicating enhanced aggregation, while all ILs exhibited structure-making effects in water. Antimicrobial activity was evaluated against three Gram-positive and three Gram-negative bacterial species, four mold species, two yeast species, and five bacteriophage species. The ILs showed substantially greater antimicrobial activity than nicotine. Antibacterial activity increased with alkyl chain length, and longer-chain ILs outperformed a commercial disinfectant, benzalkonium chloride, in selected antibacterial and antiviral assays. Antifungal activity was comparable to or slightly lower than that of benzalkonium chloride, depending on the species. *N*-tetradecylnicotinium bromide exhibited the strongest antiviral activity. These results demonstrate a clear relationship between IL structure, aggregation behavior, hydration properties, and antimicrobial activity.

## 1. Introduction

**Figure 1:**
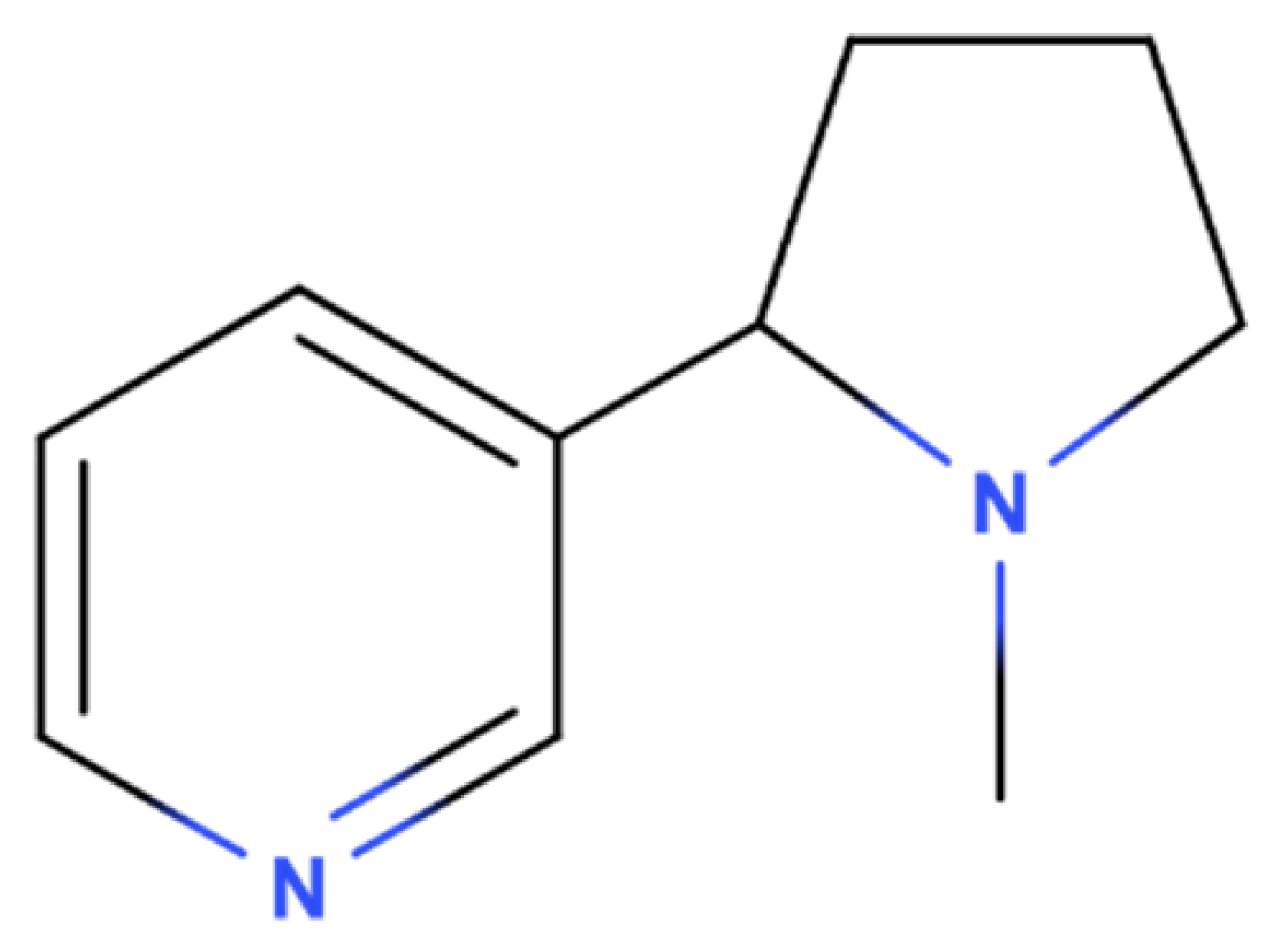
Structure of nicotine.

Nicotine is a naturally occurring pyridine alkaloid (Figure 1) widely recognized for its recreational use as a stimulant and its reported anxiolytic effects through e-cigarettes and tobacco products. The use of nicotine leads to addiction in humans, and prolonged consumption can cause serious health issues, including cancer. Nicotine’s psychoactive effects result from its ability to bind to nicotinic acetylcholine receptors in the central nervous system, causing stimulation and, at high doses, overstimulation and potential death [1]. In nature, it is found in several species of plants within the *Solanaceae* family, most notably in the tobacco plant (*Nicotiana tabacum*), and in smaller amounts in eggplants, tomatoes, and potatoes [2,3]. Due to its impact on animal nervous systems, it is believed that nicotine’s natural role is as a pesticide, and it has historically been used for this purpose in agriculture [4]. However, the non-selective toxicity of this molecule has led to its ban as a pesticide. Some derivatives of nicotine, known as neonicotinoids, continue to be used as pesticides, though they face mixed approval from legislative bodies [5].

Nicotine contains a tertiary pyrrolidine nitrogen that can be targeted for alkylation, forming a quaternary ammonium cation. Quaternary ammonium salts (QAS), compounds with an ammonium cation bonded to four organic groups and a counter anion (e.g., Cl^−^, Br^−^) [6], are known for their lytic properties. The positively charged ammonium group readily attaches to the negatively charged surfaces of cell walls and membranes. Subsequently, the hydrophobic alkyl chain penetrates the lipid bilayer, destabilizing the membrane and eventually causing rupture, which leads to cell lysis. This mechanism endows QAS with broad-spectrum antimicrobial activity, particularly against Gram-positive and Gram-negative bacteria, as well as some fungi and viruses [6]. QAS are widely used as disinfectants. However, they have limitations. Microbes lacking lipid envelopes, such as polio, adenovirus, or some bacteriophages, are not sensitive to these compounds [7].

Even more intriguing are compounds that exhibit properties of QAS and, at the same time, ionic liquids (IL). Ionic liquids (ILs) are salts made entirely of ions that are liquid below 100 °C or even at room temperature. They usually consist of a bulky organic cation (such as imidazolium, pyridinium, or ammonium derivatives) and various anions (e.g., halides, tetrafluoroborate) [8]. Because of their low volatility, high thermal stability, and adjustable properties, ILs find use in many applications. Their chemical structure can be modified to achieve specific characteristics. Biologically, ionic liquids also exhibit interesting antimicrobial activity. Some display antibacterial and antifungal properties [9]. The properties of ionic liquids, such as their ability to be mixed with buffers and their resistance to crystallization, make them very convenient for biological experiments. However, many ILs with long alkyl chains can be cytotoxic, so it is essential to design a suitable structure and verify the biocompatibility of the synthesized material [10].

The most common modification of QAS is attaching a long alkyl chain. Many reports show that increasing the length of the alkyl chain increases the antimicrobial properties of the compound. Soukup et al. [11] described many newly synthesized mono-cationic ammonium salts. The authors show a connection between the alkyl chain length and antimicrobial behavior. Compounds containing long alkyl chains (C > 12) exhibit the most potent antimicrobial activity, as the increased chain length enhances hydrophobicity and the ability to rupture cell membranes [12]. New compounds based on *N*-alkyl pyridine show antibacterial properties similar to benzalkonium chloride against some bacteria [13]. This shows that pyridinium cations with alkyl chains are valuable compounds for combating bacteria. Some QAS can also fight against viruses—cetylpyridinium and benzalkonium chloride neutralize influenza, herpes, coronaviruses, and other enveloped viruses by destroying their lipid envelope [14]. However, this activity is limited and cannot be broadly used.

The existing literature reports that several nicotine-based ionic liquids have been synthesized and examined. Kaur et al. [15] examined chiral ionic liquids based on (S)-nicotine for enantiomeric recognition. Alishahi et al. [16] examined the use of nicotine-based ILs as catalysts for benzothiazole synthesis. Vroulias et al. [17] studied the use of nicotine-based ILs in membranes for gas dehumidification. Nobuoka and Kitaoka [18] examined the use of nicotine-based ILs for metal ion-responsive gelation. Singh et al. [19] reported nicotine-based, ester-functionalized, surface-active ILs and examined their self-assembly and cytotoxicity.

This work presents a series of nicotine derivative-based ILs, *N*-alkylnicotinium bromides, with alkyl side chains ranging from octyl to tetradecyl. Apart from *N*-octylnicotinium bromide, previously reported by Vroulias et al. [17] as an intermediary compound, these ILs yield novel surface-active compounds that, to the best of our knowledge, have not been previously investigated. The hydration properties of these ILs were examined by measuring density, sound velocity, viscosity, and conductivity of their aqueous solutions. The antibacterial, antifungal, and antiviral properties of these compounds were also examined due to their suspected high antimicrobial activity as both surface-active and quaternary ammonium compounds. Hydration properties were strongly influenced by the self-assembly of the ILs into micelles, with alkyl-chain length decreasing the CMC. It was found that all four compounds are notable antibacterial, antifungal, and antiviral agents, highlighting the potential of nicotine-based ionic liquids as biologically active materials derived from natural compounds.

## 2. Results and Discussion

### 2.1. Critical Micellar Concentration—Conductometry

Due to the surface-active nature of substances with long alkyl side chains, we examined micelle formation by conductometric determination of the critical micellar concentration (CMC). The determination of CMC was performed by measuring the conductivity of a series of IL aqueous solutions of known concentrations at room temperature (25 °C). The data collected for the conductivity of IL aqueous solutions are presented in Table S1 and shown graphically in Figure S1.

As shown in Figure S1, conductivity increases with IL concentration. However, the plots can be divided into two regions of linear growth with different slopes. The point where the slope changes indicates the CMC. Below the CMC, individual surfactant monomers are fully ionized and can move freely, conducting charge more easily. Beyond the CMC, micelles form, with a different charge-to-mass ratio, leading to less efficient charge transfer and a slower increase in conductivity with concentration. The CMC value is determined by finding the intersection point of the extrapolated lines from the two linear regions of the plot [20,21]. The CMC values are listed in Table 1. These values decrease as the alkyl side chain length increases, which is typical for amphiphilic surfactants [22].

The decrease in CMC with longer alkyl chains indicates stronger hydrophobic interactions and a greater tendency toward self-assembly. These amphiphilic traits are also likely to affect biological activity, as antimicrobial quaternary ammonium compounds mainly work by inserting hydrophobic chains into microbial membranes.

### 2.2. Volumetric Measurements

The antimicrobial activity of amphiphilic ionic liquids is closely related to their physicochemical behavior in aqueous media. Properties such as micellization, hydration, and intermolecular interactions influence how these molecules interact with biological membranes. Therefore, understanding the aggregation behavior and solvation of nicotine-derived ionic liquids provides insight into their biological activity.

The densities of aqueous solutions of all four nicotine ILs were measured and are shown in Table S2 and visually represented in Figure S2 in the Supplementary Materials. In all four systems, density decreases as IL molality increases and as temperature rises. We used the density values to calculate the apparent molar volumes (*V*_φ_) following Equation (1), which are listed in Table S2.

(1) $$V_{\phi }=\frac{1000\left(d_{1}-d\right)}{{\mathrm{mdd}}_{1}}+\frac{M}{d}$$

where *d* and *d*_1_ are the densities of the solution and pure solvent, respectively; *M* is the molar mass of the dissolved IL, and *m* is the molality of the solution. Further, by linear fitting the molality to *V*_φ_ in accordance with Masson’s equation for electrolytes [23] (Equation 3), we obtained the apparent molar volume at infinite dilution (*V*_φ_°) as the intercept and the experimental slope (*S*_v_). The values of (*V*_φ_°) and *S*_v_ are given graphically in Figure S3 and also in Table 2 with standard deviations (*σ*) and regression coefficients (*R*^2^) for the linear fit.

(2) $$V_{\phi }=V_{\phi }{}^{\circ}+S_{v}\sqrt{m}$$

From Table S2 and Figure S3, we observe that *V*_φ_ for all four IL systems increases with IL molality, temperature, and alkyl side-chain length. The fitting parameters of Masson’s equation are useful for understanding the interactions present in the aqueous solutions of the examined ILs. The slope *S*_v_ shows the change in volume with the addition of solute; the greater the absolute value, the more the change in volume depends on the solute concentration, and it is often used as an indicator of solute–solute interactions [24,25]. The positive slope value *S*_v_ in all four IL aqueous systems indicates that the solution volume increases with increasing IL content. The intensity of this change, as measured by the absolute value of *S*_v_, differs among the four ILs. Generally, *S*_v_ values increase as the alkyl chain length increases. This could be explained by the hydrophobicity of the ILs and the increasing affinity for self-aggregation as the chain lengthens, as shown by the CMC values. *S*_v_ is often interpreted as an indicator of solute–solute interactions, and as all four ILs form micelles, the difference in *S*_v_ values can be correlated neatly with the ease of micelle formation. With C_14_NicBr, a very small concentration of the IL is needed for micelles to begin forming, which is reflected in a rapid change of *V*_Φ_ with IL molality. C_8_NicBr, on the other hand, has the highest CMC value and requires a significantly greater concentration for micelles to form; therefore, the change in volume with IL addition is less intense. *S*_v_ values for all four IL systems also generally increase with increasing temperature, with only C_14_NicBr being an exception, with a generally decreasing trend of *S*_v_ with *T*. CMC values of surfactants are temperature-dependent, and that dependency is often found to have a U-shape, with a characteristic minimum of CMC at a certain temperature (*T*_min_) [26,27]. While the determination of CMC in an expanded temperature range is beyond the scope of this work, the literature shows that there is a trend for longer-chain surfactants to have a lower *T*_min_ [28,29]. Therefore, the decreasing value of *S*_v_ with temperature could indicate that the *T*_min_ value of C_14_NicBr is at the start of or below the examined temperature range for density, and it is reflected in lower solute–solute interaction intensities, as shown by *S*_v_. This hypothesis should, however, be examined in further work dedicated to the self-aggregation of these ILs in water.

Using the calculated data for apparent molar volumes at infinite dilution and fitting their temperature dependence with a second-order polynomial (Equation (3)), we obtain the coefficients *a*_0_, *a*_1_, and *a*_2_.

*V_ϕ_*° = *a*_0_ + *a*_1_*T* + *a*_2_*T*^2^ (3)

The coefficients, shown in Table S3, are further used to calculate limiting molar expansibility (*E*_φ_°):

(4) $$E_{\phi }{}^{\circ}={\left(\frac{\partial V_{\phi }{}^{\circ}}{\partial T}\right)}_{p}=a_{1}+2a_{2}T$$

Table S4 shows the calculated values of *E*_φ_° for all four examined IL systems, which are graphically compared in Table 3.

*E*_φ_° reflects the change of *V*_φ_° with temperature; it provides insight into how temperature affects a system’s ability to expand. There is no clear dependence of expansibility on alkyl chain length, generally decreasing from C_8_NicBr to C_12_NicBr but again increasing significantly with C_14_NicBr. Additionally, temperature dependence is also not uniform; C_8_NicBr and C_14_NicBr have decreasing *E*_φ_° values with temperature, while C_10_NicBr and C_12_NicBr have increasing values with temperature. Therefore, expansibility is not governed solely by alkyl chain length. A potential explanation is the differences between ionic and hydrophobic hydration. The particularly pronounced behavior of C_14_NicBr may be associated with its larger hydrophobic region, leading to both a recruitment of a larger amount of water molecules into its hydration sphere and the rapid loss of these solvent molecules with temperature, as they are bound by weaker interactions.

The coefficients from Equation (4) can also be used to calculate Hepler’s coefficient via the following equation [30]:

(5) $${\left(\frac{\partial E_{\phi }{}^{\circ}}{\partial T}\right)}_{p}={\left(\frac{\partial^{2}V_{\phi }{}^{\circ}}{\partial T^{2}}\right)}_{p}=2a_{2}.$$

The values of Hepler’s coefficient with standard error and *p*-values are given in Table 4.

Hepler’s coefficient is usually associated with structure-making and structure-breaking properties for aqueous solutions, depending on the sign of the coefficient. However, it has several weaknesses that make it unreliable. One apparent weakness, demonstrated in Table 4, is that its values are often both near zero and have significant errors due to the number of equations that separate it from measured data. As *p*-values are >0.05, and the confidence interval for all four covers 0, we do not regard Hepler’s coefficient as a useful indicator for structure making/breaking in this work.

### 2.3. Acoustic Measurements

Measurements of sound velocity in all four IL systems were taken, and the values are shown in Table S5 and visually represented in Figure S4 in the Supplementary Materials. The speed of sound increases in all four IL systems with both molality and temperature.

Speed of sound values can be further utilized to calculate isentropic compressibility of both the solution (*κ*_s_) and pure solvent (*κ*_s,2_) through Equations (6) and (7).

(6) $$\kappa_{s}=\frac{1}{u^{2}d}$$

(7) $$\kappa_{s,2}=\frac{1}{u_{2}^{2}d_{2}}$$

where *u* is the speed of sound in the solution, *u*_2_ is the speed of sound in pure solvent, while d is the density in the solution, and *d*_2_ is the density in pure solvent. The calculated values of *κ*_s_ and *κ*_s,2_ are shown in Table S5. Compressibility values are further utilized to calculate apparent molar isentropic compressibility (*κ*_φ_) in accordance with the following equation:

(8) $$\kappa_{\phi }=\left[\frac{\kappa_{s}d_{2}-\kappa_{s,2}d}{{\mathrm{mdd}}_{2}}\right]+\kappa_{s}\frac{M_{1}}{d}$$

where *M*_1_ is the molar mass of the given IL and *m* is the molality of the solution. The value of *κ*_φ_° can be used in Masson’s equation for electrolytes to calculate the apparent molar isentropic compressibility at infinite dilution (*κ*_φ_°):

*κ_ϕ_* = *κ_ϕ_*° + *S_k_m*^1/2^. (9)

The obtained values of (*κ*_φ_°) and the experimental slope *S*_k_ are given in Table 5.

Values of apparent molar adiabatic compressibility at infinite dilution (*κ*_φ_°) increase with temperature for all four IL systems, starting from negative values and becoming positive at higher temperatures for C_8_NicBr, C_10_NicBr, and C_12_NicBr. These negative values indicate the presence of “rigid” interactions that resist compression and could be attributed to these systems still being predominantly hydrated monomers, as indicated by their CMC values. Compressibility increases with temperature as the system’s resistance to compression decreases with heightened thermal motion and therefore becomes more pliable [31].

The results for the experimental slope *S*_k_ is very similar to results of the slope *S*_v_ for volumetric data; the slope *S*_k_ can be assumed to be an indicator of solute–solute interactions [32]. The results for Sk are in very good agreement with the results for *S*_v_. C_14_NicBr has the highest value, which decreases with decreasing alkyl chain length. Therefore, the *S*_k_ values support and can be interpreted in the same way that the *S*_v_ values have been.

### 2.4. Viscometric Measurements

Viscosity of the IL aqueous solutions was measured, and the results are shown in Table S6 and graphically displayed in Figure S5 in the Supplementary Materials.

To better understand the influence of these ILs on water structure, viscosity values were further used in accordance with the Jones–Dole equation for electrolytes [33]:

(10) $$\frac{\eta }{\eta_{{}^{\circ}}}=1+Ac^{1/2}+\mathrm{Bc},$$

where *A* and *B* are coefficients, *η* is the viscosity of the solution, *η*_o_ is the viscosity of the pure solvent, and c is the concentration of nicotine in the ionic liquids. Equation (11) can be simplified to the following linear form, where coefficient *B* is the slope of the linear fit. Values of the *B* coefficient are given in Table 6.

(*η*/*η_o_* − 1)/*c*^1/2^ = *A* + *Bc*^1/2^ (11)

The Jones–Dole coefficient *B* is a useful criterion for determining the structure-making/breaking characteristics of a solute. Positive values of the coefficient indicate structure-making behavior. As shown in Table 6, all four examined IL solutions exhibit relatively large and positive *B* coefficients, indicating significant interactions with the surrounding water. This is not unusual, as ions with long hydrophobic alkyl chains often have positive *B* coefficients, attributed to the effect of hydrophobic hydration on the surrounding water [34]. Additionally, the values are overall in good agreement with the literature data on *B* coefficient values of surface-active ILs [35], with the exception of C_10_NicBr, which has an abnormally large B coefficient. However, this outlier behavior of the *B* coefficient of a C10 derivative has also been noted in the literature [35].

### 2.5. Antimicrobial Activity

The physicochemical measurements show that increasing the alkyl chain length improves hydrophobic interactions, reduces the CMC, and promotes aggregation in water. These structural modifications enhance the amphiphilic nature of the molecules, which is known to be crucial for the antimicrobial activity of quaternary ammonium compounds, as they disrupt microbial membranes.

#### 2.5.1. Antibacterial Activity

The results of the antibacterial activity of nicotine and synthesized nicotine-derived ILs against three Gram-negative (G-) bacteria (*Escherichia coli* ATCC 8739, *Pseudomonas aeruginosa* ATCC 9027, and *Proteus mirabilis* ATCC 12453) and three Gram-positive (G+) bacteria (*Micrococcus luteus* JR-10, *Staphylococcus aureus* ATCC 25923, and *Staphylococcus epidermidis* JR-07) are presented in Table S7 in the Supplementary Materials.

Bacteria were the most sensitive to all synthesized ILs (the MIC and MBC ranged from 1.53·10^−6^ to 7.81·10^−4^ mol/dm^3^), while they were the least sensitive to nicotine (MIC and MBC: 2.5·10^−2^ mol/dm^3^ in Gram-positive; 5.0·10^−2^ mol/dm^3^ in Gram-negative). Both nicotine and nicotine-derived ILs showed a stronger effect on Gram-positive bacteria compared to Gram-negative bacteria (Table S7). This trend has also been observed in studies of other types of ILs [36,37,38] due to the more complex cell wall structure of Gram-negative bacteria, which results in weaker sensitivity and permeability [39,40,41].

**Figure 2:**
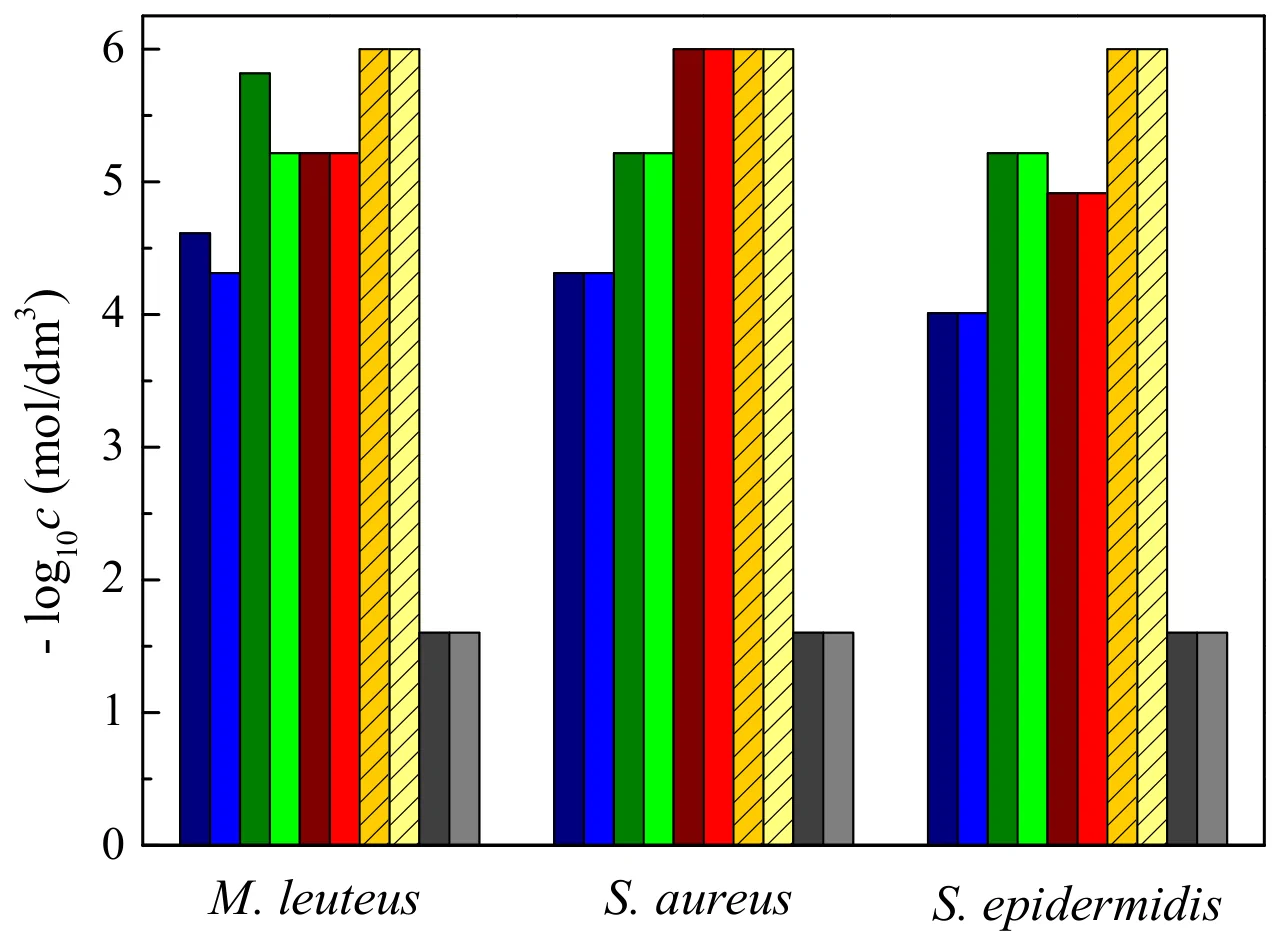
MIC (darker) and MBC (lighter) values against Gr+ bacteria of ■ C_8_NicBr, ■ C_10_NicBr, ■ C_12_NicBr, ■ C_14_NicBr, and ■ nicotine. Patterned bars are for results below the lowest examined concentrations and are used only for easier overview and do not represent concrete numerical values.

Unlike nicotine, which had a uniform effect on different species within the G+/G- bacteria group, the examined ILs displayed a more specific impact. Among G+ bacteria, the effect of ILs decreased in the following order: *M. luteus*, *S. aureus*, and *S. epidermidis*, with *M. luteus* being the most sensitive of all tested bacteria, responding to some of the lowest concentrations tested (Figure 2).

**Figure 3:**
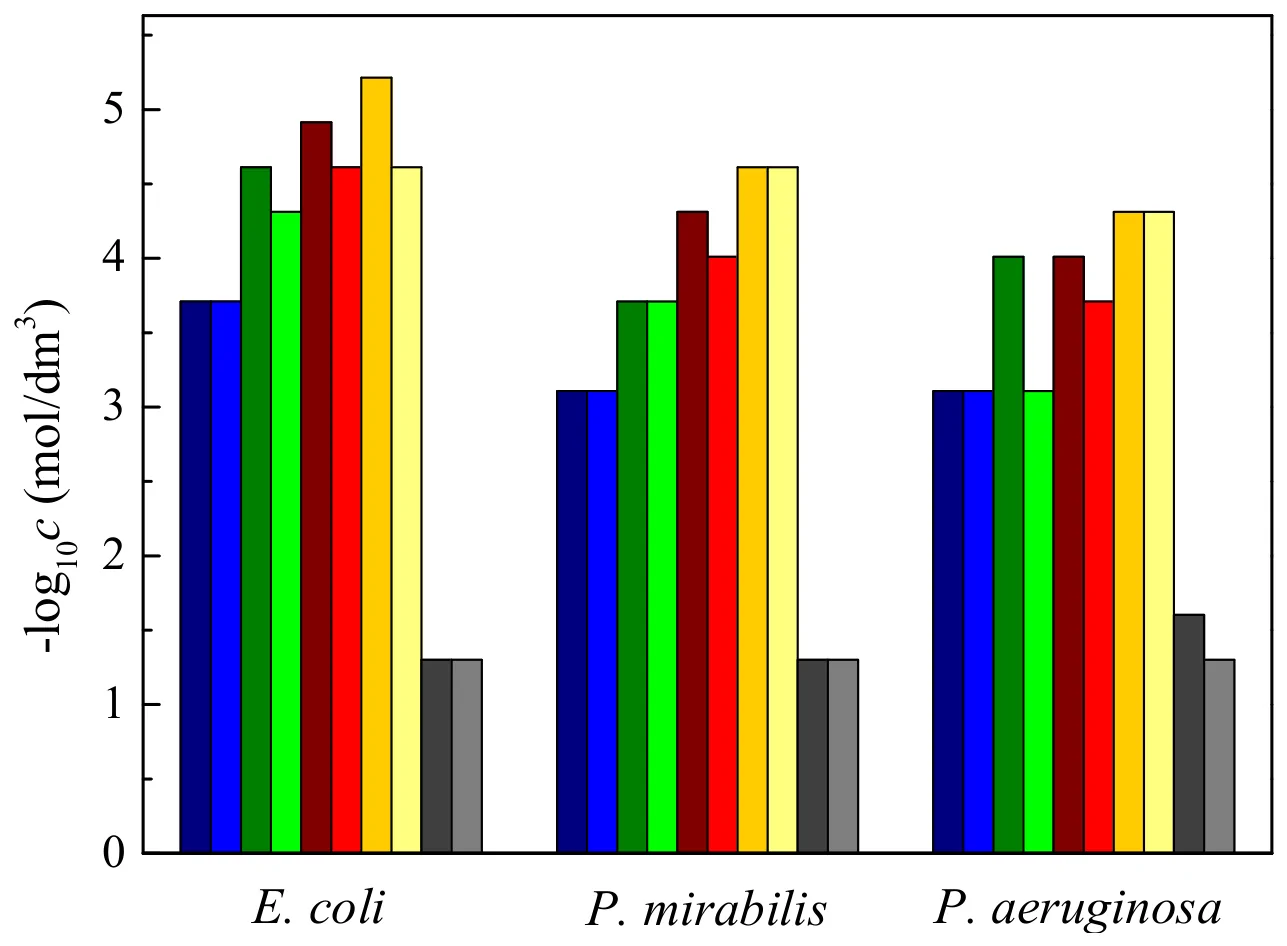
MIC (darker) and MBC (lighter) values against Gr− bacteria of ■ C_8_NicBr, ■ C_10_NicBr, ■ C_12_NicBr, ■ C_14_NicBr, and ■ nicotine.

*E. coli* was the most sensitive Gram-negative bacterium to all synthesized ILs, while *P. aeruginosa* was the most resistant of all bacteria (Figure 3). This aligns with previous reports indicating that *E. coli* is quite sensitive to various new antimicrobial agents, unlike *P. aeruginosa*, a member of the ESCAPE group of multidrug-resistant pathogens, which tends to exhibit a high level of resistance to different antimicrobial agents [42,43,44,45].

In both groups of bacteria, increasing the number of carbon atoms in the alkyl chain of ILs from 8 to 14 enhances antimicrobial activity. Therefore, the most effective IL was C_14_NicBr, which inhibited the growth of all bacteria at the lowest concentrations, while the MIC and MBC values for Gram-positive bacteria were even lower than the tested concentrations. This IL also has the highest hydrophobicity, which may further boost its antimicrobial activity [41,46]. Previous studies on the impact of different alkyl chain lengths on the antimicrobial activity of various ILs have also shown that ILs with alkyl chains of 12 or 14 carbon atoms exhibit the highest antimicrobial activity [10,38,47,48], while a significant decrease in activity was observed for chains with fewer than 10 or more than 16 carbons [49]. Compared to other studies, our nicotine derivatives demonstrated an antibacterial effect 50 to 400 times stronger than tetrabutylphosphonium-based ILs with medium-chain (C_6_-C_12_) fatty acids as anions [38] and 20 to 50 times stronger than imidazolium ILs with medium-length alkyl side chains on the cation (up to C_10_) and dicyanamide as the anion [48]. This suggests that adding an alkyl side chain to the cation is more effective at increasing antibacterial activity than adding it to the anion. This trend may be due to the shape of our ILs’ cations, which are more optimal for membrane penetration because of their hydrophilic positively charged heads and hydrophobic tails, which closely resemble phospholipids. Furthermore, in our study, the newly synthesized ILs showed significantly greater antimicrobial activity than their precursor, nicotine.

Compared to the literature values of a commercially used antimicrobial disinfectant, benzalkonium chloride (BenCl), the newly synthesized ILs, specifically the longer chain C_12_NicBr and C_14_NicBr, showed superior antibacterial activity to BenCl against *E. coli* [50], *P. aeruginosa* [51], and *S. aureus* [52,53], with MBC values of the ILs lower by 2 to 30 times. Values of MICs and MBCs from the literature are shown in Table S7.

#### 2.5.2. Antifungal Activity

The results of antifungal activity of nicotine and synthesized nicotine-derived ILs against two yeasts (*Candida albicans* ATCC 10231 and *Candida guilliermondii* JR-23) and four filamentous fungi (*Penicillium verrucosum* FCD0025, *Penicillium chrysogenum* FCD0035, *Aspergillus flavus* FCD0049, and *Aspergillus parasiticus* FCD0050) are presented in Table S7.

Compared to all test microorganisms, *P. verrucosum* and yeasts were the most sensitive to the effects of nicotine, although its MIC and MFC values were still much higher (from 3.12·10^−3^ to 6.25·10^−3^ mol/dm^3^) compared to the effective concentrations of ILs (MIC/MFC: from 2.4·10^−5^ to 1.56·10^−3^ mol/dm^3^). As in the case of bacteria, the antifungal activity of nicotine was significantly enhanced by the synthesis of nicotine-based ILs.

**Figure 4:**
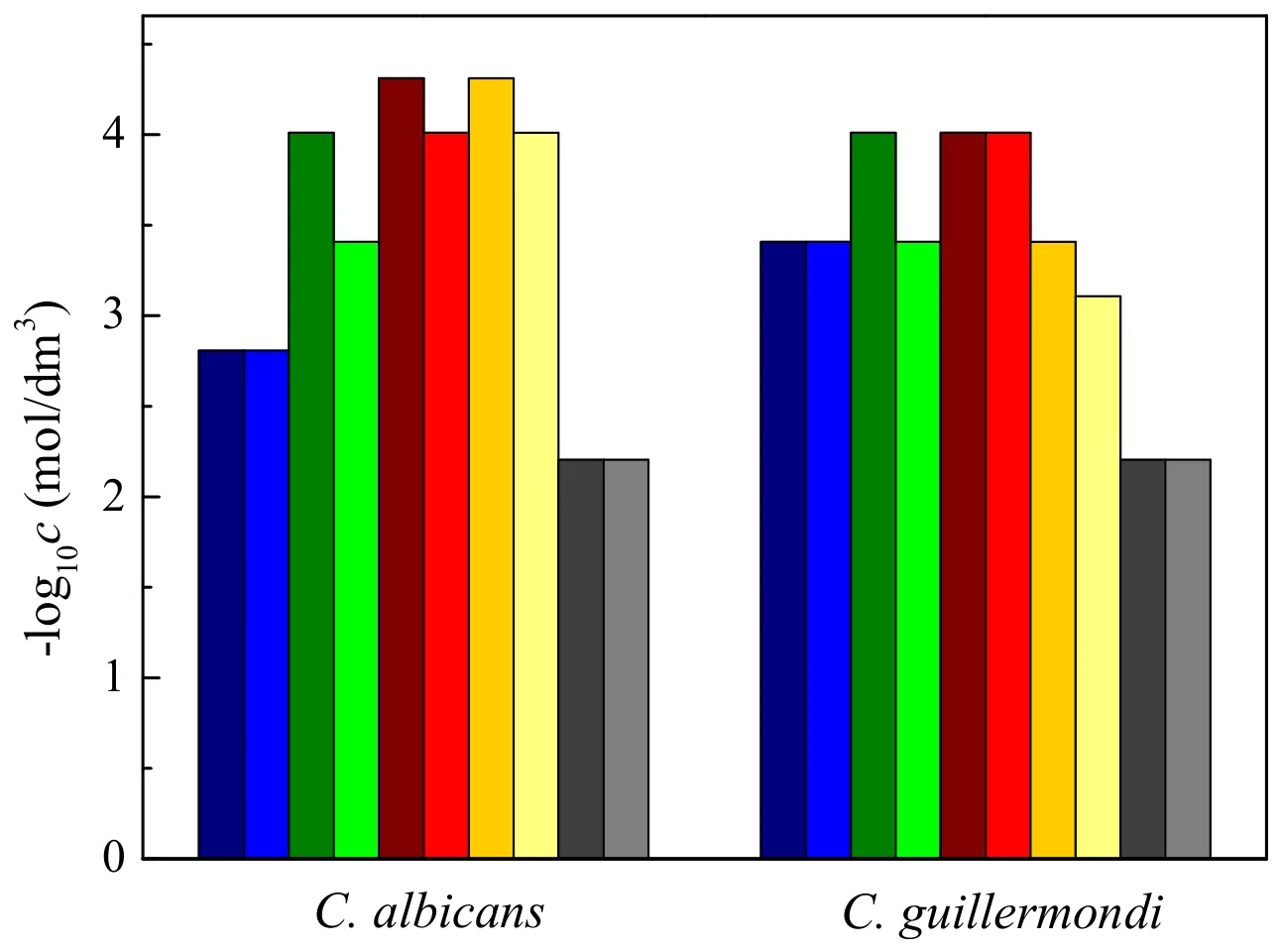
MIC (darker) and MBC (lighter) values against yeasts of ■ C_8_NicBr, ■ C_10_NicBr, ■ C_12_NicBr, ■ C_14_NicBr, and ■ nicotine.

Only in the case of yeasts did the examined ILs fail to show a characteristic trend of increasing antimicrobial activity with longer side alkyl chains (Figure 4). The most effective ILs were C_10_NicBr and C_12_NicBr. C_8_NicBr was the weakest agent against *C. albicans*. *N*-tetradecylnicotinium bromide had the weakest antimicrobial activity against *C. guilliermondii* compared to other ILs, while its activity against *C. albicans* remained the same as that exhibited by *N*-dodecylnicotinium bromide. These results suggest that the newly synthesized nicotine-derived IL with a 12-carbon side chain is a critical point beyond which antimicrobial activity against *Candida* species begins to level off. A similar pattern was previously observed for tetrabutylphosphonium-based ILs against these two *Candida* species, with a C_10_ side chain length identified as the critical point [38].

**Figure 5:**
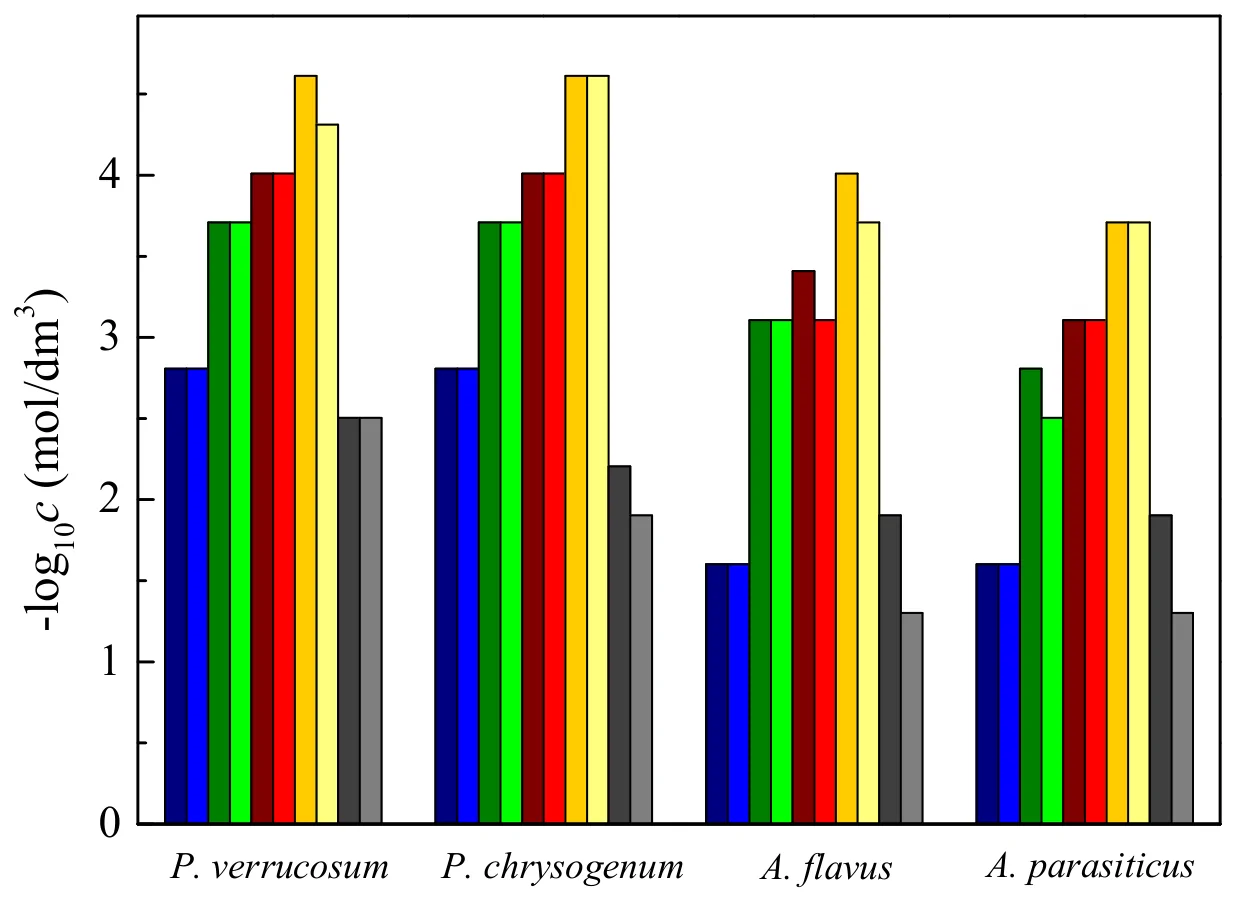
MIC (darker) and MBC (lighter) values against filamentous fungi of ■ C_8_NicBr, ■ C_10_NicBr, ■ C_12_NicBr, ■ C_14_NicBr, and ■ nicotine.

Unlike yeasts, and similar to other studied microorganisms, the sensitivity of molds to newly synthesized ILs increased with longer alkyl side chains (Figure 5). Molds belonging to the genus *Aspergillus* were the most resistant of all tested microorganisms to both nicotine and nicotine-based ILs. Contrary to our results, in the work of Panić et al. [38], tetrabutylphosphonium-based ILs with medium-chain fatty acids as anions showed similar antimicrobial effects against Gram-negative bacteria (the same species as in our study), yeasts (except *C. albicans*), and molds (the same species as in our study, except *A. parasiticus*).

Compared to the literature values of BenCl, the newly synthesized ILs, specifically the longer-chain C_12_NicBr and C_14_NicBr, exhibited slightly weaker antifungal activity against *C. albicans*, with 4.88·10^−5^ mol/dm^3^ for the ILs versus 0.6–2.3·10^−5^ mol/dm^3^ for BenCl [51]. Meanwhile, BenCl was notably more effective against both *A. flavus* and *A. parasiticus* [54]. Values of MIC and MFC from the referenced literature are shown in Table S7.

#### 2.5.3. Antiviral Activity

**Figure 6:**
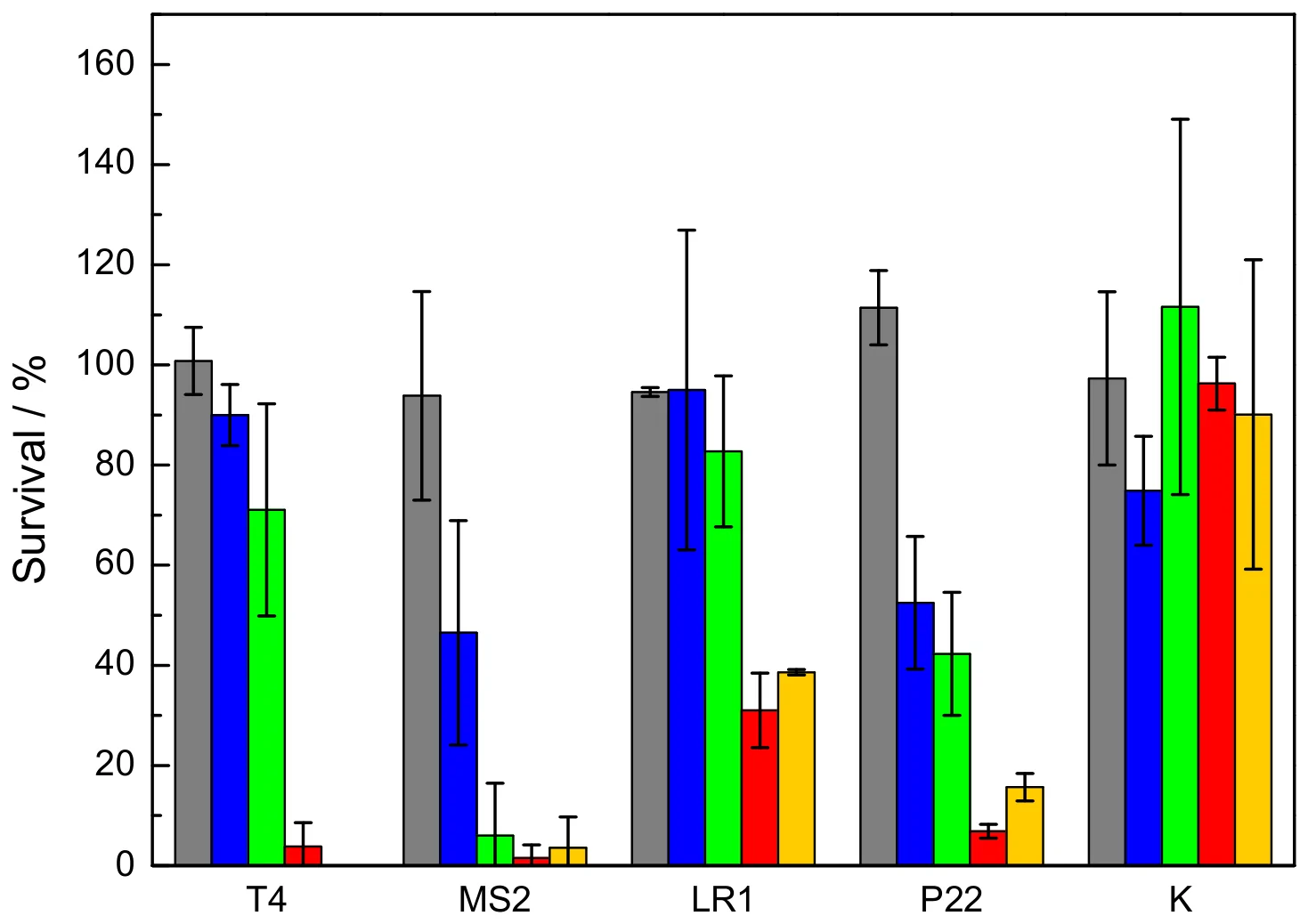
Survival rate values of tested bacteriophages against ■ nicotine, ■ C_8_NicBr, ■ C_10_NicBr, ■ C_12_NicBr, and ■ C_14_NicBr.

We decided to study the effects of nicotinic ILs on a judiciously chosen set of bacteriophages. Phages are (1) often used as surrogates of pathogenic viruses, and thus finding a way to fight them can be translated to healthcare and agricultural applications [55,56], and (2) they cause substantial losses in the biotech industry, where bacteria are used for the production of active compounds [57]. First, we tested T4 (host *E. coli*), a representative of tailed phages, which constitute the majority of known bacteriophages [58]. MS2 (host *E. coli*) is considered a difficult target for antiviral compounds [59]. LR1 (host *P. aeruginosa*) is an example of an environmental phage (isolated from the Baltic Sea), and P22 (host *Salmonella enterica* serovar Typhimurium) is related to the food industry. P22 is also considered a difficult target for antivirals [60]. Phage K (broad host range, primarily infecting a wide array of *Staphylococcus aureus* strains) was the only tested phage infecting G+ bacteria. All studied phages lack lipid membranes. As mentioned before, QAS are less efficient against targets without a lipid envelope [7]. The results of the antiviral assay of the four nicotinic ILs show that the sensitivity of bacteriophages to the tested compounds varies greatly depending on the type of phage (Figure 6).

The strongest antiviral effects were observed against phages T4 and MS2. Complete inactivation was observed for T4 incubated with C_14_NicBr, while MS2 was completely inactivated by C_10_NicBr, C_12_NicBr, and C_14_NicBr (detection limit around 25 PFU/ML). Since T4 is a tailed phage, attachment of the amphiphilic cation to the capsid or tail fibers might cause structural changes or damage that prevent the phage from injecting DNA, explaining this phage’s susceptibility to the tested ILs. For MS2, the literature suggests that positively charged QAS readily bind to capsids, causing virion aggregation by neutralizing their surface charge and thus preventing infection [61].

Some inactivation of phage LR1 was observed. Moderate decreases in infectious activity were recorded for C_12_NicBr and C_14_NicBr, with survival rates of 32% and 44%, respectively. Shorter chains (C_8_NicBr and C_10_NicBr) did not affect the LR1 titer. It has been reported that *P. aeruginosa* phages are resistant to benzalkonium chloride, which might explain the moderate activity of our ILs [62,63]. Phage P22 showed greater susceptibility to the tested ILs, especially for C_12_NicBr and C_14_NicBr, with survival rates of 5% and 12%, respectively.

For LR1 and P22, antiviral activity did not increase with longer alkyl side chains; the highest inactivation was achieved with C_12_NicBr, which consistently exhibited greater antiphage activity than C_14_NicBr. Long alkyl chains may cause micelle formation or self-aggregation of the cation, reducing the availability of monomeric disinfectant molecules to interact with the virion. Although the tested concentration was below the bulk CMC, surface-induced aggregation or adsorption-driven local enrichment near virion surfaces cannot be excluded. In contrast, C_12_NicBr has a CMC roughly four times higher and might offer an optimal balance, being sufficiently hydrophobic to disrupt phage particles but not so effective as to cause inactivation [64]. Additionally, Zhu [65] highlighted the importance of the hydrophobic linker length on the properties of antiviral compounds.

Phage K (broad host range, primarily infecting a wide array of *Staphylococcus aureus* strains) showed the greatest resistance, with no significant decrease in survival observed for any of the tested derivatives. In the staphylococcal systems, cationic nicotinium derivatives could be scavenged by anionic cell-wall material present in the lysate, especially wall teichoic acids (WTA). WTA is abundant in *S. aureus* and serves as a receptor for staphylococcal phages; thus, WTA-rich residues may bind part of the compound before it reaches the virion surface, thereby lowering the effective antiviral dose around phage K [66,67]. It was found that benzalkonium chloride did not significantly affect the *Staphylococcus* phage at bacteriostatic concentrations [68]. Our observations are in line with these reports.

Overall, it can be concluded that among the tested viruses, bacteriophages LR1, P22, and K were difficult to inactivate with the tested compounds, while *E. coli* phages (T4, MS2) were effectively neutralized, especially by longer alkyl derivatives. No effect of unmodified nicotine was observed on any of the tested phages. The results align with the literature data for benzalkonium chloride (BenCl), also a QAS, which is commonly used as a disinfectant [7,69]. Also, the susceptibility of the various tested phages to our ILs followed the literature data for BenCl—LR1, a *P. aeruginosa* phage, showed only moderate deactivation [62,63], whereas phage K (*S. aureus* phage) was not affected at all [70].

#### 2.5.4. Antiviral Comparison Test Results

BenCl is typically used at concentrations of 0.1–0.5%, corresponding to about 1·10^−2^ mol·dm^−3^, to achieve complete inactivation. In this study, we aimed to determine whether C_14_NicBr, the best overall performer among the four tested ILs, outperforms the commercially used BenCl, even at much lower concentrations, ranging from 1·10^−4^ to 1·10^−10^ mol·dm^−3^.

**Figure 7:**
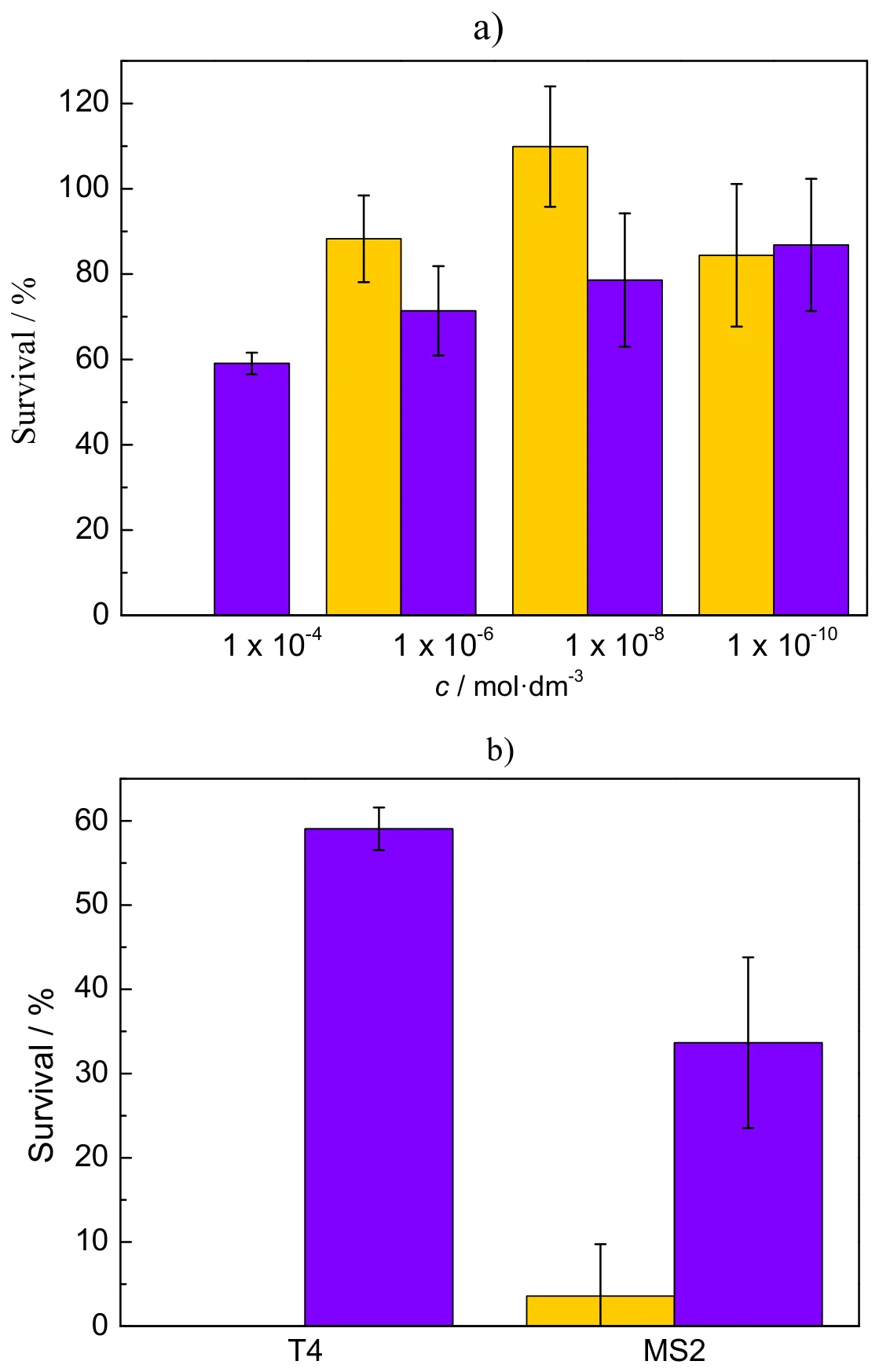
Comparison of the effect of ■ C_14_NicBr and ■ BenCl on the survival of (**a**) bacteriophage T4 at concentrations of 10^−4^, 10^−6^, 10^−8^, and 10^−10^ mol·dm^−3^, and (**b**) bacteriophages T4 and MS2 at a concentration of 10^−4^ mol·dm^−3^.

Firstly, we evaluated the effect on phage T4 (Figure 7a). C_14_NicBr showed significantly greater inactivation at 1·10^−4^ mol·dm^−3^ than BenCl. Lower concentrations of both C_14_NicBr and BenCl did not significantly affect T4 (both samples were similar to the control). Since there was no meaningful antiviral effect of either substance below 1·10^−4^ mol·dm^−3^, only the concentration of 1·10^−4^ mol·dm^−3^ was tested for comparison against phage MS2 (Figure 7b). The results showed that C_14_NicBr reduced the MS2 titer by 100% (no visible plaques), while BenCl at the same concentration reduced the titer by about 75%. Although both substances exhibited notable antiviral effects, the complete inactivation by the nicotinic IL compared with the partial inactivation by BenCl indicates that the IL is a much more potent antiviral agent. This finding is especially interesting because MS2 is a non-enveloped virus, which is typically quite resistant to disinfectants [69].

## 3. Materials and Methods

### 3.1. Materials

Nicotine (CAS number: 54-11-5, purity 99%) was obtained from Sigma Aldrich (Sigma-Aldrich Chemie GmbH, Steinheim, Germany). 1-bromooctane (CAS number 111-83-1, purity 99%) and 1-bromotetradecane (CAS number 112-71-0, purity 97%) were acquired from Sigma Aldrich (Sigma-Aldrich Chemie GmbH, Steinheim, Germany). 1-bromodecane (CAS number 112-29-8, purity 98%) and 1-bromododecane (CAS number 143-15-7, purity 98%) came from Acros Organics (Fisher Scientific (Austria) GmbH, Wien, Austria). Chloroform (CAS number 67-66-3, purity 99.9%) was purchased from Lachner (Lach-Ner, s.r.o., Neratovice, Czech Republic), and diethyl ether (CAS number 60-29-7, purity 100%) was obtained from VWR Chemicals (VWR International, Leuven, Belgium). All chemicals were used as received without further purification.

### 3.2. Synthesis

Synthesis of all four ionic liquids was performed using the same procedure. Nicotine and the appropriate alkyl bromide were mixed in a 1:1.05 molar ratio (nicotine:alkylbromide) with approximately 20 mL of chloroform as the solvent in a balloon flask. The mixture was then heated to 90 °C under reflux for 72 h. During the synthesis of all four ILs, the initial bright yellow color of the mixture turned dark reddish-brown as the reaction progressed. Afterward, chloroform was removed from the reaction flask using a rotary evaporator. Excess reactants were removed via extraction with diethyl ether. This extraction was repeated five times, and the remaining diethyl ether was removed with a rotary evaporator. Finally, the flask was placed in a vacuum dryer at 40 °C for 24 h.

**Figure 8:**
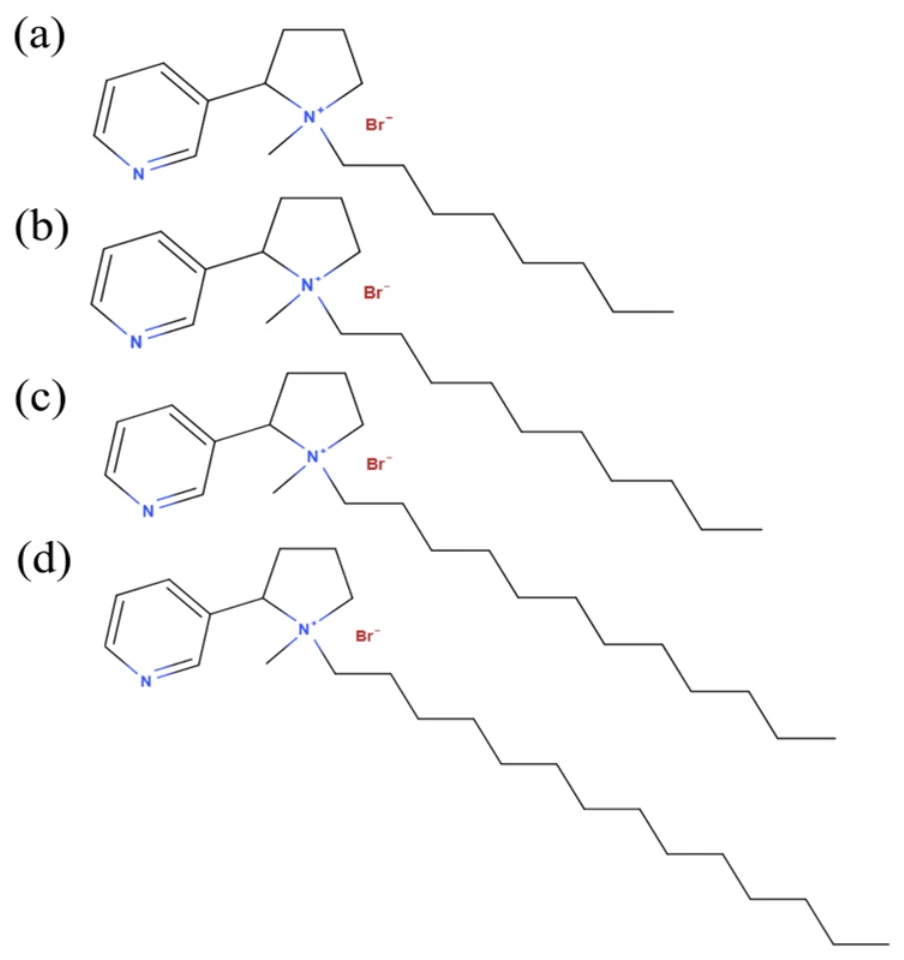
Structures of: (**a**) *N*-octylnicotinium bromide, (**b**) *N*-decylnicotinium bromide, (**c**) *N*-dodecylnicotinium bromide, and (**d**) *N*-tetradecylnicotinium bromide.

The four nicotine ionic liquids obtained are *N*-octylnicotinium bromide (C_8_NicBr), *N*-decylnicotinium bromide (C_10_NicBr), *N*-dodecylnicotinium bromide (C_12_NicBr), and *N*-tetradecylnicotinium bromide (C_14_NicBr). Proposed structures of these ionic liquids are shown in Figure 8. NMR confirmed the structures of the synthesized ILs, with the exception of the chiral conformation at the quaternary nitrogen. All ionic liquids were in a liquid state and remained liquid in the entire temperature range examined. Water content was determined via Karl–Fischer Titration to be below 500 ppm for all four ILs. Synthesis had a high level of mass yield (>91%), and all losses are attributed to diethyl ether purification and loss during transfer between flasks.

### 3.3. Measurement Apparatus and Procedures

NMR spectra were recorded in deuterium oxide (D_2_O) at 25 °C using a Bruker Avance III 400 MHz instrument (Bruker Corp, Billerica, Massachusetts, USA). Tetramethylsilane served as an internal standard for calibrating the chemical shifts of ^1^H and ^13^C spectra. The ^13^C spectra were obtained through a selective decoupling technique. To interpret the spectra, ^1^H homodecoupling and 2D HSQC methods were employed. Both ^1^H and ^13^C spectra and their assignments are provided in the Supplementary Materials in Figure S6 and Table S8.

Measurements of density, sound velocity, and viscosity were performed on aqueous solutions of examined ionic liquids in a range of molalities from 0.02 to 0.10 mol·kg^−1^ for C_8_NicBr, from 0.001 to 0.005 mol·kg^−1^ for C_10_NicBr, from 0.0009 to 0.005 mol·kg^−1^ for C_12_NicBr, and from 0.0005 to 0.0025 mol·kg^−1^ for C_14_NicBr. Measurements were performed at 5 temperatures (293.15, 298.15, 303.15, 308.15, and 313.15 K). Molality ranges were determined based on CMC values determined using conductimetry; all tested ranges were below their respective CMC values.

Density and sound velocity measurements of the ILs were carried out using a vibrating tube densimeter equipped with a sound measuring cell, Anton Paar DSA 5000 M (Anton Paar GmbH, Graz, Austria). Thermal control in the device was maintained with a built-in thermostat with ±0.001 K accuracy.

Viscosity of the ionic liquid solutions was measured using an Ostwald viscometer (Xylem Analytics GmbH, Weilheim, Germany, type no. 530 03 capillary viscometer). A ViscoClock plus (Xylem Analytics GmbH, Weilheim, Germany,) timing unit regulated the measurements. The viscometer was held within a sheath filled with distilled water, with thermal control provided by a Lauda DLK 10 thermostat (Lauda Dr. R. Wobser GmbH & Co. Lauda-Königshofen, Germany). Each measurement was performed only after the bath temperature (±0.01 K) was maintained for 15 min, allowing the sample to reach thermal equilibrium. Flow-time measurements were repeated five times with a maximum deviation of 1%. Dynamic viscosity (*η*) was calculated according to the following equation:

(12) $$\eta =d\cdot \left(L\cdot t-\frac{K}{t^{2}}\right)$$

where *K* and *L* are the constants of the viscometer, determined by calibration with water (*K* = 47.669 mm^2^ s, *L* = 1.0729·10^−4^ mm^2^ s^−2^), d is the density of the solution, and *t* is the flow time.

Conductivity was measured using a Mettler-Toledo SevenExcellence (Mettler-Toledo International Inc. Greifensee, Switzerland) multimeter with a Mettler-Toledo InLab 731-ISM (Mettler-Toledo International Inc. Greifensee, Switzerland) conductivity probe. Samples of aqueous IL solutions with known molality were placed in a thermally controlled, double-walled flask with a temperature stability of ±0.1 K, maintained by a Lauda E111 thermostat (Lauda Dr. R. Wobser GmbH & Co. Lauda-Königshofen, Germany). After 15 min at constant temperature, the probe was inserted and the conductivity was measured.

### 3.4. Antimicrobial Tests

#### 3.4.1. Antibacterial and Antifungal Tests

Antimicrobial activity of the four ionic liquids and unmodified nicotine was assessed in vitro in triplicate using a microdilution method in sterile 96-well microtiter plates. The methodology followed the adapted CLSI/EUCAST guidelines [70,71,72]. The following standard reference and laboratory strains were used: three Gram-negative bacteria (Gr−): *Escherichia coli* (ATCC 8739), *Proteus mirabilis* (ATCC 12453) and *Pseudomonas aeruginosa* (ATCC 9027); three Gram-positive bacteria: *Micrococcus luteus* (JR10), *Staphylococcus aureus* (ATCC 25932), and *Staphylococcus epidermidis* (JR07); two yeasts: *Candida albicans* (ATCC 10231) and *Candida guilliermondii* (JR23); and four molds: *Penicillium verrucosum* (FCD0025), *Penicillium chrysogenum* (FCD0035), *Aspergillus flavus* (FCD0049), and *Aspergillus parasiticus* (FCD0050). Microbial strains were pre-cultured on Mueller–Hinton agar (Torlak, Belgrade, Serbia) (bacteria, at 37 °C) or malt extract agar (yeasts at 37 °C, molds at 26 °C). Bacterial and yeast inocula were adjusted to 0.5 McFarland and diluted (in Mueller-Hinton/malt broth) to obtain final concentrations of 1–2·10^5^ CFU/mL (bacteria) and 1–5·10^5^ CFU/mL (yeasts). Mold spore suspensions were prepared in saline solution, adjusted to 1.5·10^6^ spores/mL in the Bürker Türk (Torlak, Belgrade, Serbia) counting chamber, and were used to inoculate malt broth to a final concentration of 1.5·10^5^ CFU/mL. Each test well contained equal volumes (100 μL) of IL solution (double dilution in sterile water) and inoculated broth. ILs were tested over a concentration range of 0.05 mol·dm^−3^ to 1.53·10^−6^ mol/dm^3^. After an incubation period (24 h at 37 °C for bacteria, 48 h at 37 °C for yeasts, and 72 h at 26 °C for molds), the growth in each well was compared to the growth control well, which lacked antimicrobial agents, as well as to a broth-sterility control, which had no microorganisms added. MIC values were determined visually as the lowest IL concentration at which no visible growth occurred. For MBC/MFC determination, aliquots from test wells at and above the MIC were subcultured on agar plates (Mueller–Hinton agar/malt extract agar) and incubated under the same conditions. The lowest IL concentration, reducing viability by 99.9%, was recorded as the MBC or MFC.

#### 3.4.2. Antiviral Tests

Antiviral tests were performed on five species of bacteriophages: T4 (*Tequatrovirus T4*, Straboviridae), and MS2 (*Emesvirus zinderi*, Fiersviridae), which were purchased from Phage Consultants, Gdańsk Poland; LR1_PAO1 (likely the Lindbergviridae, isolated from the environment), P22 (*Lederbergvirus P22*, family unassigned, purchased from Leibniz Institute DSMZ-German Collection of Microorganisms and Cell Cultures GmbH, Leibniz Germany), and K (*Kayvirus kay*, Herelleviridae, purchased from ATCC, Maassas, Virginia, USA). MS2 has positive-sense single-stranded RNA ((+)ssRNA). Other tested phages contain a genome as double-stranded DNA (dsDNA). T4 is a large tailed phage infecting *E. coli* (the BL21 strain was used as a host) and represents the structure of a typical dsDNA bacteriophage. MS2 is a small, tailless phage with an icosahedral capsid that also infects *E. coli* (C3000 strain). MS2 is often used as a model for non-enveloped viruses [73,74]. LR1 is a tailed phage infecting *Pseudomonas aeruginosa* (PAO1, DSM 22644) and was isolated from Baltic Sea water from the Gulf of Gdańsk. P22 is an icosahedral phage infecting *Salmonella enterica* (DSM 18522) and is known for its high resistance to chemical agents. Phage K is a large tailed phage specific for Gram-positive bacteria, infecting *S. aureus* (ATCC 19685).

Viruses (with an initial titer of about 10^6^ PFU/mL) were mixed with solutions of the tested derivatives (at a concentration of 10^−4^ mol·dm^−3^ if not stated otherwise) and incubated for 3 h at room temperature. The titer was then determined using the double overlay method. Briefly, Petri plates were first prepared with 5 mL of LB-agar medium. Then, 5 mL of top agar mixed with 200 µL of refreshed host bacterial culture was poured onto the plates. Appropriate dilutions of the phage samples were prepared. Eight 5 µL droplets were placed onto the top agar layer. After a 24 h incubation at 37 °C, the plaques were counted and converted to plaque-forming units per milliliter (PFU/mL). The results presented in Figure 7 and Figure 8 are expressed as means ± standard deviation (SD) from three independent biological replicates (*n* = 3).

LB medium’s composition was 10 g/L of NaCl, 10 g/L of tryptone, and 5 g/L of yeast extract. For experiments, 10 g of powder was dissolved in 400 mL of water and then sterilized in an autoclave. For LB-agar, 6 g of agar was added to the LB medium; for top-agar, 2 g of agar was added. The media were ordered from Carl Roth (Carl Roth GmbH + Co KG, Karlsruhe, Germany). TM buffer (pH 7.4) was prepared by mixing 10 mM Tris base, 5 μM CaCl_2_, 10 mM MgSO_4_, and deionized water (Mili-Q water purification system, Merck KGaA, Darmstadt, Germany). All media were autoclaved before use.

#### 3.4.3. Antiviral Comparison Test

Once determined, the best-tested IL would undergo a comparison test against a standard disinfectant; in this case, benzalkonium chloride (BenCl). A comparison was made between two model phages, T4 and MS2, which were previously shown to be significantly inactivated by the tested ILs. The effectiveness was checked at different concentrations: 10^−4^ mol·dm^−3^, 10^−6^ mol·dm^−3^, 10^−8^ mol·dm^−3^, and 10^−10^ mol·dm^−3^ for phage T4, and at a concentration of 10^−4^ mol·dm^−3^ for phage MS2. The incubation time was again 3 h.

## 4. Conclusions

In this work, we successfully synthesized and characterized a series of nicotine-based ionic liquids, *N*-alkylnicotinium bromides with alkyl chain lengths from C_8_ to C_14_. Their physicochemical behavior in aqueous solutions was studied through conductometric, volumetric, acoustic, and viscosimetric measurements, and their antimicrobial potential was evaluated against a wide range of bacteria, fungi, and bacteriophages.

Conductometric measurements showed a clear dependence of the critical micellar concentration (CMC) on alkyl chain length, with CMC values dropping significantly as the hydrophobic chain length increased. This behavior indicates increased amphiphilic nature and stronger hydrophobic interactions of the longer-chain ionic liquids, which encourage self-assembly into micellar structures in water. Volumetric and acoustic parameters further supported this, revealing differences in hydration and intermolecular interactions across the series. Viscosimetric analysis confirmed strong solute–solvent interactions and the structure-making nature of the ionic liquids in water, characteristic of amphiphilic electrolytes.

Antimicrobial investigations showed that converting nicotine into quaternary ammonium ionic liquids greatly enhances its biological activity. The produced ionic liquids demonstrated strong antibacterial and antifungal effects, with bacteria being the most sensitive and molds the most resistant. The ILs also exhibited potent antiviral activity against bacteriophages T4 and MS2. Overall, antimicrobial effectiveness increased with longer alkyl side chains; C_14_NicBr showed the strongest overall performance, particularly in antibacterial and selected antiviral assays. Even when compared to a standard disinfectant, benzalkonium chloride, C_14_NicBr displayed higher antibacterial and antiviral performance, while its antifungal activity was mostly similar to BenCl.

Taken together, the results highlight a clear relationship between the structure of nicotine-based ionic liquids, their aggregation and hydration behavior in aqueous solution, and their antimicrobial activity. Increasing the alkyl chain length enhances amphiphilicity, strengthens hydrophobic interactions, and promotes micellar self-assembly, all of which contribute to more effective interactions with microbial membranes and heightened antimicrobial activity. These findings demonstrate that nicotine-based ionic liquids are a promising class of biologically active, amphiphilic materials and that their physicochemical properties can be adjusted through structural modification to optimize antimicrobial performance. However, many ILs with long alkyl chains can be cytotoxic or ecotoxic if not properly designed. Therefore, a key direction for future research is to evaluate the cytotoxicity of *N*-tetradecylnicotinium bromide on mammalian cells and its biodegradability in the environment.

## Figures and Tables

**Table 1:** Values of CMC for all four examined IL aqueous solutions.

System	CMC/mol·dm^3^
Aqueous solution of *N*-octylnicotinium bromide	0.1007
Aqueous solution of *N*-decylnicotinium bromide	0.0397
Aqueous solution of *N*-dodecylnicotinium bromide	0.0086
Aqueous solution of *N*-tetradecylnicotinium bromide	0.0022

**Table 2:** Fitting parameters were obtained using Masson′s equation (Equation (3)) for electrolytes in all four aqueous solutions of the ILs over the temperature range from *T* = (293.15 to 313.15) K, with standard deviations (*σ*) and regression coefficients (*R*^2^).

**Aqueous solution of C_8_NicBr**				
*T*/K	*S*_v_/cm^3^·kg·mol^−2^	*V*_ϕ_°/cm^3^ mol^−1^	*σ*/cm^3^ mol^−1^	*R* ^2^
293.15	7.04	298.56	0.0429	0.99213
298.15	8.56	299.79	0.0730	0.9847
303.15	8.79	301.33	0.0788	0.9831
308.15	12.86	301.70	0.1056	0.9858
313.15	14.22	303.03	0.1197	0.9850
**Aqueous solution of C_10_NicBr**				
*T*/K	*S*_v_/cm^3^·kg·mol^−2^	*V*_ϕ_°/cm^3^ mol^−1^	*σ*/cm^3^ mol^−1^	*R* ^2^
293.15	317.8	300.77	0.7401	0.9774
298.15	336.4	300.89	1.068	0.9586
303.15	346.7	301.94	0.9287	0.9703
308.15	358.0	305.50	1.110	0.9605
313.15	361.8	306.94	1.005	0.9681
**Aqueous solution of C_12_NicBr**				
*T*/K	*S*_v_/cm^3^·kg·mol^−2^	*V*_ϕ_°/cm^3^ mol^−1^	*σ*/cm^3^ mol^−1^	*R* ^2^
293.15	302.6	338.98	1.0625	0.9518
298.15	311.7	339.68	0.6714	0.9814
303.15	313.8	342.34	0.7957	0.9744
308.15	328.3	342.94	0.5268	0.9896
313.15	316.1	346.55	0.8667	0.9702
**Aqueous solution of C_14_NicBr**				
*T*/K	*S*_v_/cm^3^·kg·mol^−2^	*V*_ϕ_°/cm^3^ mol^−1^	*σ*/cm^3^ mol^−1^	*R* ^2^
293.15	1623	309.97	3.1563	0.9721
298.15	1426	320.44	1.5526	0.9911
303.15	1159	335.95	2.0436	0.9769
308.15	1064	343.30	2.0723	0.9720
313.15	1231	339.95	1.8923	0.9824

**Table 3:** Values of *E*_φ_° for all four IL systems at all measured temperatures.

*E*_φ_°/cm^3^·mol^−1^·K^−1^					
*T*/K	293.15 K	298.15 K	303.15 K	308.15 K	313.15 K
aq. *N*-octylnicotinium bromide	0.272	0.245	0.217	0.189	0.162
aq. *N*-decylnicotinium bromide	0.046	0.193	0.339	0.485	0.632
aq. *N*-dodecylnicotinium bromide	0.153	0.261	0.368	0.476	0.583
aq. *N*-tetradecylnicotinium bromide	3.703	2.680	1.656	0.633	−0.390

**Table 4:** Values of Hepler’s coefficient for all four examined IL systems.

System	Hepler’s Coefficient	*p*
Aqueous solution of *N*-octylnicotinium bromide	−0.0055 ± 0.0072	0.5235
Aqueous solution of *N*-decylnicotinium bromide	0.0293 ± 0.0184	0.2509
Aqueous solution of *N*-dodecylnicotinium bromide	0.0215 ± 0.0172	0.3375
Aqueous solution of *N*-tetradecylnicotinium bromide	−0.2047 ± 0.0777	0.1190

**Table 5:** Values of apparent molar adiabatic compressibility at infinite dilution (*κ*_φ_°) and *S_k_* as determined according to Equation (11) for all four IL aqueous solutions in the temperature range *T* = (293.15 to 313.15) K, with standard deviations (*σ*) and regression coefficients (*R*^2^).

**Aqueous solution of *N*-octylnicotinium bromide**				
*T*/K	*S*_k_*·*10^14/^ m^3^·mol^−3/2^·Pa^−1^·kg^−1^	*κ*_φ_°·10^14/^ m^3^·mol^−1^·Pa^−1^	*σ·*10^14^/m^3^·mol^−1^·Pa^−1^	*R* ^2^
293.15	6.61	−4.10	0.1303	0.9220
298.15	6.33	−2.65	0.1202	0.9275
303.15	6.08	−1.32	0.0932	0.9516
308.15	6.97	−0.42	0.1127	0.9465
313.15	6.82	0.60	0.0634	0.9818
**Aqueous solution of *N*-decylnicotinium bromide**				
*T*/K	*S*_k_*·*10^14/^ m^3^·mol^−3/2^·Pa^−1^·kg^−1^	*κ*_φ_°·10^14/^ m^3^·mol^−1^·Pa^−1^	*σ·*10^14^/m^3^·mol^−1^·Pa^−1^	*R* ^2^
293.15	34.86	−5.40	0.0569	0.9888
298.15	15.21	−1.24	0.0699	0.9678
303.15	15.59	0.13	0.0923	0.9465
308.15	−21.06	2.36	0.0890	0.9267
313.15	−88.54	7.77	0.5181	0.9469
**Aqueous solution of *N*-dodecylnicotinium bromide**				
*T*/K	*S*_k_*·*10^14/^ m^3^·mol^−3/2^·Pa^−1^·kg^−1^	*κ*_φ_°·10^14/^ m^3^·mol^−1^·Pa^−1^	*σ·*10^14^/m^3^·mol^−1^·Pa^−1^	*R* ^2^
293.15	33.76	−4.37	0.1014	0.9644
298.15	46.31	−1.83	0.1208	0.9730
303.15	31.09	0.82	0.1511	0.9106
308.15	36.26	1.94	0.1456	0.9376
313.15	28.16	3.26	0.0750	0.9718
**Aqueous solution of *N*-tetradecylnicotinium bromide**				
*T*/K	*S*_k_*·*10^14/^ m^3^·mol^−3/2^·Pa^−1^·kg^−1^	*κ*_φ_°·10^14/^ m^3^·mol^−1^·Pa^−1^	*σ·*10^14^/m^3^·mol^−1^·Pa^−1^	*R* ^2^
293.15	614.7	−28.42	1.2919	0.9675
298.15	549.6	−21.75	0.8230	0.9833
303.15	322.5	−9.45	0.7035	0.9650
308.15	637.3	−22.88	1.6811	0.9494
313.15	410.0	−11.09	1.0327	0.9529

**Table 6:** Values of the Jones–Dole coefficient *B* for nicotine IL aqueous solutions in the temperature range: *T* = (293.15 to 313.15) K.

*B*/dm^3^*·*mol^−1^				
*T*/(K)	C_8_NicBr	C_10_NicBr	C_12_NicBr	C_14_NicBr
293.15	1.582	9.56	2.340	4.358
298.15	1.490	10.89	2.678	2.399
303.15	1.390	16.18	1.998	4.414
308.15	1.396	14.40	2.793	5.447
313.15	1.345	18.22	2.336	5.766

## Data Availability

The data supporting this article have been included as part of Supplementary Materials.

## Supplementary Materials

The following supporting information can be downloaded at: https://www.mdpi.com/article/10.3390/molecules31214001/s1, Table S1: change of conductivity (*κ*) with change of molality for all four examined ILs (*m*), Table S2: Density (*d*), apparent molar volume (*V*_ϕ_), and partial molar volume of solute (${\bar{V}}_{2}$) and solvent (${\bar{V}}_{1}$) at different temperatures and molality of all four examined ionic liquids (m), Table S3: The values of the *a*_0_, *a*_1_ and *a*_2_ coefficients for all four examined IL aqueous solutions, Table S4: Values of apparent molar expansibility for all four IL aqueous solutions, Table S5: Sound velocity (u), isentropic compressibilities of the solution (*κ*_s_) and apparent molar isentropic compressibility (*κ*_Φ_) at different temperatures and molality (*m*) for all four IL aqueous solutions, Table S6: Viscosity (*η*), reduced viscosity (*η*/*η*_o_), concentration (*c*) and reduced viscosity (*η*/*η*_o_) at different temperatures and molality (*m*) for all four ILs., Table S7: The MIC and MBC values (expressed in mol·dm^−3^) for nicotine and the four nicotine-based ionic liquids on all 12 examined microorganisms, alongside comparisons against BenCl from literature [1,2,3,4,5] and additional values expressed in mM and mg/L, Table S8: ^1^H and ^13^C NMR spectra assignments for all four ionic liquids, Figure S1: Dependence of conductivity *κ* versus molality *m* of aqueous solutions of: (a) C_8_NicBr, (b) C_10_NicBr (c) C_12_NicBr, (d) C_14_NicBr, Figure S2: Variation of density of aqueous solutions of: (a) C_8_NicBr; (b) C_10_NicBr (c) C_12_NicBr (d) C_14_NicBr with molality at different temperatures: ● 293.15; ● 298.15; ● 303.15; ● 308.15; ● 313.15 K. Figure S3: Linear fit of the square of molality of: (a) C_8_NicBr; (b) C_10_NicBr (c) C_12_NicBr (d) C_14_NicBr with apparent molar volume (*V*_Φ_) as per Masson’s equation, at different temperatures: ● 293.15; ● 298.15; ● 303.15; ● 308.15; ● 313.15 K. Figure S4: Variation of speed of sound in aqueous solutions of: (a) C_8_NicBr; (b) C_10_NicBr (c) C_12_NicBr (d) C_14_NicBr with molality at different temperatures: ● 293.15; ● 298.15; ● 303.15; ● 308.15; ● 313.15 K. Figure S5: Variation of viscosity in aqueous solutions of: (a) C_8_NicBr; (b) C_10_NicBr (c) C_12_NicBr (d) C_14_NicBr with molality at different temperatures: ● 293.15; ● 298.15; ● 303.15; ● 308.15; ● 313.15 K. Figure S6: (a) ^1^H and (b) ^13^C NMR spectrum for ■ *N*-octylnicotinium bromide ■ *N*-decylnicotinium bromide; ■ *N*-dodecylnicotinium bromide; ■ *N*-tetradecylnicotinium bromide

## Abbreviations

The following abbreviations are used in this manuscript:

IL	Ionic Liquid
SAIL	Surface Active Ionic Liquid
QAS	Quaternary Ammonium Salt
C_8_NicBr	*N*-octylnicotinium bromide
C_10_NicBr	*N*-decylnicotinium bromide
C_12_NicBr	*N*-dodecylnicotinium bromide
C_14_NicBr	*N*-tetradecylnicotinium bromide
CMC	Critical Micellar Concentration
MIC	Minimal Inhibitory Concentration
MBC	Minimal Bactericidal Concentration
MFC	Minimal Fungicidal Concentration
BenCl	Benzalkonium Chloride
