# Supplementary material for: Synthesis, Hydration, Antibacterial, Antifungal, and Antiviral Properties of New Surface-Active Nicotine-Based Ionic Liquids
Source: Molecules. 2026 Nov 6;31(21):4001. doi: 10.3390/molecules31214001 (PMC13764947; doi:10.3390/molecules31214001)
Supplement: Supplementary file 1 [file molecules-31-04001-s001.zip › molecules-4598023-supplementary.pdf]

Synthesis, Hydration, Antibacterial, Antifungal, and Antiviral Properties of New Surface-Active Nicotine-Based Ionic Liquids

Andrija Vukov [1], Milana Rakić [2], Maja Karaman [2], Bartosz Kamiński [3], Jan Paczesny [3], Janez Cerar [4] and Milan Vraneš [1,*]

[1] Department of Chemistry, Biochemistry and Environmental Protection, Faculty of Sciences, University of Novi Sad, Trg Dositeja Obradovića 3, 21000 Novi Sad, Serbia; andrija.vukov@dh.uns.ac.rs
[2] Department of Biology and Ecology, Faculty of Sciences, University of Novi Sad, Trg Dositeja Obradovića 3, 21000 Novi Sad, Serbia; milana.novakovic@dbe.uns.ac.rs (M.R.); maja.karaman@dbe.uns.ac.rs (M.K.)
[3] Institute of Physical Chemistry, Polish Academy of Sciences, Marcina Kasprzaka 44/52, 01-224 Warsaw, Poland; bkaminski@ichf.edu.pl (B.K.); jpaczesny@ichf.edu.pl (J.P.)
[4] Faculty of Chemistry and Chemical Technology, University of Ljubljana, Večna pot 113, 1000 Ljubljana, Slovenia; janez.cerar@fkkt.uni-lj.si
* Correspondence: milan.vranes@dh.uns.ac.rs

Table S1. change of conductivity (κ) with change of molality for all four examined ILs (m).

aq. N-octylnicotinium bromide		aq. N-dodecylnicotinium bromide	
m / mol·kg^{-1}	κ / mS·cm^{-1}	m / mol·kg^{-1}	κ / mS·cm^{-1}
0.005	0.480465	0.001997	0.203003
0.01	0.904157	0.003	0.277843
0.02	1.709303	0.003994	0.378816
0.03	2.466008	0.005	0.461129
0.04	3.178090	0.006	0.529031
0.05	3.909502	0.007	0.637698
0.0625	4.764136	0.007987	0.745212
0.075	5.285844	0.0096	0.800298
0.08	5.668314	0.0125	0.887014
0.09	6.069648	0.015	0.96634
0.1	6.642437	0.019968	1.120426
0.125	8.052671	0.025032	1.256130
0.15	9.030662	0.03	1.387849
0.2	11.06743	0.032929	1.464453
0.25	12.21564	/	/
0.3	13.08588	/	/
aq. N-decylnicotinium bromide		aq. N-tetradecylnicotinium bromide	
m / mol·kg^{-1}	κ / mS·cm^{-1}	m / mol·kg^{-1}	κ / mS·cm^{-1}
0.00275	0.321615	0.00025	0.024850
0.0055	0.520405	0.0005	0.048237
0.00825	0.712427	0.001	0.086926
0.011	0.986709	0.00125	0.107506
0.022	1.855387	0.0015	0.122110
0.0275	2.483691	0.002	0.157738
0.033	2.527520	0.0025	0.182473
0.0396	2.921532	0.002675	0.193447
0.055	3.443823	0.0029	0.201212
0.066	3.795297	0.0034	0.218406
0.06875	3.834137	0.004	0.244598
0.0825	4.050610	0.005	0.276959
0.11	4.897696	0.006	0.313428
0.1375	5.449599	0.007	0.342321
0.165	6.178876	0.008	0.377266
/	/	0.01	0.443416

Table S2. Density (d), apparent molar volume (V_ϕ), and partial molar volume of solute ($\overline{V}_2$) and solvent ($\overline{V}_1$) at different temperatures and molality of all four examined ionic liquids (m).

m / mol·kg^{-1}	d / g·cm^{-3}	$\overline{V}_1$ / cm^3·mol^{-1}	$\overline{V}_2$ / cm^3·mol^{-1}	V_ϕ / cm^3·mol^{-1}
**N-octylnicotinium bromide**				
T = 293.15 K				
0.02000	0.999348	18.05	300.01	299.54
0.04091	1.000485	18.05	300.66	299.98
0.05996	1.001496	18.05	301.15	300.33
0.07999	1.002542	18.05	301.53	300.58
0.09987	1.003567	18.05	301.80	300.74
T = 298.15 K				
0.02000	0.998165	18.07	301.57	300.98
0.04091	0.999276	18.07	302.32	301.48

0.05996	1.000257	18.07	302.98	301.96
0.07999	1.001273	18.07	303.44	302.26
0.09987	1.002272	18.07	303.73	302.42
T = 303.15 K				
0.02000	0.996745	18.10	303.13	302.54
0.04091	0.997829	18.10	303.95	303.10
0.05996	0.998788	18.10	304.58	303.56
0.07999	0.999778	18.10	305.07	303.88
0.09987	1.000755	18.10	305.34	304.02
T = 308.15 K				
0.02000	0.995119	18.13	304.33	303.46
0.04091	0.996180	18.13	305.58	304.33
0.05996	0.997115	18.13	306.45	304.94
0.07999	0.998075	18.13	307.16	305.42
0.09987	0.999022	18.12	307.59	305.65
T = 313.15 K				
0.02000	0.993284	18.16	306.04	304.98
0.04091	0.994322	18.16	307.40	305.89
0.05996	0.995227	18.16	308.50	306.67
0.07999	0.996168	18.16	309.19	307.08
0.09987	0.997083	18.16	309.77	307.41
**N-decylnicotinium bromide**				
T = 293.15 K				
0.00108	0.998259	18.05	317.03	311.80
0.00200	0.998319	18.05	321.92	314.81
0.00258	0.998355	18.05	324.36	316.29
0.00400	0.998435	18.05	330.34	320.30
0.00519	0.998489	18.05	335.87	324.43
T = 298.15 K				
0.00108	0.997103	18.07	318.54	313.00
0.00200	0.997164	18.07	322.62	315.10
0.00258	0.997198	18.07	325.90	317.36
0.00400	0.997276	18.07	332.24	321.60
0.00519	0.997327	18.07	338.21	326.09
T = 303.15 K				
0.00108	0.995707	18.10	319.98	314.27
0.00200	0.995767	18.10	324.20	316.45
0.00258	0.995799	18.10	328.08	319.28
0.00400	0.995875	18.09	334.44	323.48
0.00519	0.995926	18.09	340.12	327.63
T = 308.15 K				
0.00108	0.994094	18.13	324.30	318.41
0.00200	0.994151	18.13	328.40	320.40
0.00258	0.994181	18.13	332.31	323.22
0.00400	0.994251	18.12	339.02	327.70
0.00519	0.994296	18.12	345.07	332.17
T = 313.15 K				

0.00108	0.992279	18.16	325.76	319.80
0.00200	0.992335	18.16	329.97	321.88
0.00258	0.992363	18.16	334.46	325.28
0.00400	0.992432	18.16	340.91	329.46
0.00519	0.992477	18.16	346.70	333.66
**N-dodecylnicotinium bromide**				
T = 293.15 K				
0.00096	0.998261	18.05	352.16	347.47
0.00202	0.998318	18.05	359.84	353.04
0.00254	0.998342	18.05	363.23	355.60
0.00403	0.998418	18.05	367.19	357.59
0.00504	0.998460	18.05	370.87	360.13
T = 298.15 K				
0.00096	0.997103	18.07	353.67	348.84
0.00202	0.997160	18.07	360.88	353.88
0.00254	0.997185	18.07	363.80	355.95
0.00403	0.997253	18.07	369.83	359.94
0.00504	0.997300	18.07	372.14	361.08
T = 303.15 K				
0.00096	0.995707	18.10	356.21	351.34
0.00202	0.995760	18.10	364.34	357.29
0.00254	0.995786	18.10	366.26	358.36
0.00403	0.995850	18.10	372.58	362.62
0.00504	0.995894	18.09	375.06	363.92
T = 308.15 K				
0.00096	0.994086	18.13	357.95	352.86
0.00202	0.994140	18.13	365.16	357.78
0.00254	0.994164	18.13	367.92	359.65
0.00403	0.994225	18.12	374.81	364.39
0.00504	0.994268	18.12	377.29	365.64
T = 313.15 K				
0.00096	0.992275	18.16	360.41	355.51
0.00202	0.992325	18.16	368.45	361.35
0.00254	0.992347	18.16	371.37	363.41
0.00403	0.992410	18.16	376.51	366.48
0.00504	0.992448	18.16	379.66	368.44
**N-tetradecylnicotinium bromide**				
T = 293.15 K				
0.00248	0.998316	18.05	429.56	389.17
0.00200	0.998305	18.05	418.78	382.50
0.00108	0.998270	18.05	392.83	366.12
0.00078	0.998254	18.05	380.46	357.81
0.00049	0.998238	18.05	360.28	342.28
T = 298.15 K				
0.00248	0.997155	18.07	427.06	391.60
0.00200	0.997149	18.07	414.73	382.87
0.00108	0.997111	18.07	392.70	369.25

0.00078	0.997096	18.07	380.60	360.71
0.00049	0.997078	18.07	366.56	350.75
T = 303.15 K				
0.00248	0.995754	18.10	423.77	394.93
0.00200	0.995751	18.10	411.26	385.35
0.00108	0.995710	18.10	395.26	376.19
0.00078	0.995697	18.10	383.76	367.58
0.00049	0.995679	18.10	374.23	361.38
T = 308.15 K				
0.00248	0.994133	18.13	424.83	398.36
0.00200	0.994132	18.13	412.20	388.42
0.00108	0.994094	18.13	396.07	378.56
0.00078	0.994080	18.13	386.81	371.96
0.00049	0.994062	18.13	379.81	368.01
T = 313.15 K				
0.00248	0.992315	18.16	432.51	401.88
0.00200	0.992313	18.16	420.61	393.10
0.00108	0.992279	18.16	403.14	382.89
0.00078	0.992268	18.16	390.99	373.81
0.00049	0.992252	18.16	380.13	366.48

Standard uncertainties are: $u(m) = 5\cdot10^{-5}$ mol·kg^{-1}, $u(T)$ = 0.015 K;
Relative standard uncertainty: $u_r(d)$ = 0.4 %, $u_r(p)$ = 1.5 %.

Table S3. The values of the a_0, a_1 and a_2 coefficients for all four examined IL aqueous solutions.

System	Coefficient	Value
**N-octylnicotinium bromide**	a_0 / cm^3·mol^{-1}	-19.213
	a_1 / cm^3·mol^{-1}·K^{-1}	1.8956
	a_2 / cm^3·mol^{-1}·K^{-2}	-0.00277
**N-decylnicotinium bromide**	a_0 / cm^3·mol^{-1}	1545.73
	a_1 / cm^3·mol^{-1}·K^{-1}	-8.541
	a_2 / cm^3·mol^{-1}·K^{-2}	0.01465
**N-dodecylnicotinium bromide**	a_0 / cm^3·mol^{-1}	1217.97
	a_1 / cm^3·mol^{-1}·K^{-1}	-6.140
	a_2 / cm^3·mol^{-1}·K^{-2}	0.01075
**N-tetradecylnicotinium bromide**	a_0 / cm^3·mol^{-1}	-9572.55
	a_1 / cm^3·mol^{-1}·K^{-1}	63.708
	a_2 / cm^3·mol^{-1}·K^{-2}	-0.10234

Table S4. Values of apparent molar expansibility for all four IL aqueous solutions.

	E_{ϕ}^{o} / cm^3·mol^{-1}·K^{-1}				
T/ K	293.15	298.15	303.15	308.15	313.15
**N-octylnicotinium bromide**	0.2725	0.2448	0.2171	0.1894	0.1618
**N-decylnicotinium bromide**	0.0461	0.1925	0.3390	0.4855	0.6319
**N-dodecylnicotinium bromide**	0.1530	0.2605	0.3681	0.4756	0.5831
**N-tetradecylnicotinium bromide**	3.7033	2.6799	1.6564	0.6330	-0.3904

Table S5. Sound velocity (*u*), isentropic compressibilities of the solution (κ_s) and apparent molar isentropic compressibility (κ_ϕ) at different temperatures and molality (*m*) for all four IL aqueous solutions

m / mol·kg^{-1}	*u* / m·s^{-1}	$\kappa_s \cdot 10^{10}$ / Pa^{-1}	$\kappa_\phi \cdot 10^{14}$ / m^3·mol^{-1}Pa^{-1}
**N-octylnicotinium bromide**			
T = 293.15 K			
0.02000	1486.92	4.526	-3.247
0.04091	1491.36	4.494	-2.574
0.05996	1495.61	4.464	-2.562
0.07999	1499.71	4.435	-2.273
0.09987	1503.60	4.407	-1.993
T = 298.15 K			
0.02000	1500.94	4.447	-1.824
0.04091	1505.00	4.418	-1.194
0.05996	1508.90	4.391	-1.180
0.07999	1512.62	4.365	-0.891
0.09987	1516.15	4.340	-0.628
T = 303.15 K			
0.02000	1512.91	4.383	-0.513
0.04091	1516.63	4.357	0.037
0.05996	1520.15	4.333	0.094
0.07999	1523.52	4.309	0.366
0.09987	1526.69	4.287	0.619
T = 308.15 K			
0.02000	1523.36	4.330	0.529
0.04091	1526.73	4.307	1.117
0.05996	1529.93	4.285	1.191
0.07999	1532.97	4.264	1.469
0.09987	1535.59	4.245	1.852
T = 313.15 K			
0.02000	1531.95	4.290	1.519

0.04091	1535.03	4.268	2.068
0.05996	1537.87	4.249	2.235
0.07999	1540.60	4.229	2.497
0.09987	1543.12	4.212	2.762
**N-decylnicotinium bromide**			
T = 293.15 K			
0.0011	1483.03	4.555	-4.246
0.0020	1483.25	4.553	-3.825
0.0026	1483.39	4.552	-3.649
0.0040	1483.73	4.550	-3.245
0.0052	1484.02	4.548	-2.851
T = 298.15 K			
0.0011	1497.06	4.475	-0.713
0.0020	1497.24	4.474	-0.642
0.0026	1497.35	4.473	-0.395
0.0040	1497.64	4.471	-0.284
0.0052	1497.90	4.469	-0.141
T = 303.15 K			
0.0011	1509.39	4.408	0.635
0.0020	1509.55	4.407	0.762
0.0026	1509.65	4.406	1.010
0.0040	1509.91	4.404	1.159
0.0052	1510.15	4.403	1.204
T = 308.15 K			
0.0011	1520.03	4.354	1.678
0.0020	1520.19	4.353	1.483
0.0026	1520.31	4.352	1.199
0.0040	1520.60	4.350	1.043
0.0052	1520.87	4.348	0.864
T = 313.15 K			
0.0011	1529.01	4.311	5.183
0.0020	1529.17	4.310	3.449
0.0026	1529.27	4.309	3.243
0.0040	1529.59	4.307	1.929
0.0052	1529.85	4.305	1.707
**N-dodecylnicotinium bromide**			
T = 293.15 K			
0.0010	1482.99	4.555	-3.342
0.0020	1483.27	4.553	-2.767
0.0025	1483.41	4.552	-2.713
0.0040	1483.79	4.549	-2.348
0.0050	1484.04	4.548	-1.893
T = 298.15 K			
0.0010	1497.04	4.475	-0.470
0.0020	1497.26	4.473	0.287
0.0025	1497.37	4.473	0.580

0.0040	1497.67	4.471	1.224
0.0050	1497.88	4.469	1.324
T = 303.15 K			
0.0010	1509.36	4.408	1.644
0.0020	1509.55	4.407	2.362
0.0025	1509.65	4.406	2.460
0.0040	1509.93	4.404	2.861
0.0050	1510.12	4.403	2.892
T = 308.15 K			
0.0010	1520.01	4.354	2.930
0.0020	1520.18	4.353	3.669
0.0025	1520.26	4.352	3.913
0.0040	1520.51	4.350	4.271
0.0050	1520.67	4.349	4.397
T = 313.15 K			
0.0010	1529.03	4.311	4.135
0.0020	1529.19	4.309	4.589
0.0025	1529.28	4.309	4.582
0.0040	1529.49	4.307	5.104
0.0050	1529.65	4.306	5.242
**N-tetradecylnicotinium bromide**			
T = 293.15 K			
0.0025	1483.23	4.553	3.460
0.0020	1483.31	4.553	-2.465
0.0011	1483.14	4.554	-7.946
0.0008	1483.06	4.555	-11.962
0.0005	1482.95	4.555	-14.108
T = 298.15 K			
0.0025	1497.26	4.473	5.144
0.0020	1497.23	4.474	2.832
0.0011	1497.14	4.474	-2.798
0.0008	1497.08	4.475	-5.925
0.0005	1497.02	4.475	-10.481
T = 303.15 K			
0.0025	1509.57	4.407	6.351
0.0020	1509.52	4.407	4.822
0.0011	1509.41	4.408	1.784
0.0008	1509.36	4.408	0.140
0.0005	1509.32	4.409	-3.118
T = 308.15 K			
0.0025	1520.18	4.353	7.752
0.0020	1520.15	4.353	5.916
0.0011	1520.11	4.353	-0.454
0.0008	1520.08	4.354	-3.884
0.0005	1520.05	4.354	-10.659
T = 313.15 K			
0.0025	1529.16	4.310	9.353

0.0020	1529.18	4.310	6.545
0.0011	1529.08	4.310	3.942
0.0008	1529.07	4.310	-0.003
0.0005	1529.02	4.311	-2.530

Standard uncertainties are: $u(m) = 5\cdot10^{-5}$ mol·kg^{-1}, $u(T) = 0.015$ K;
Relative standard uncertainty: $u_r(u) = 0.4$ %, $u_r(p) = 1.5$ %.

Table S6. Viscosity (η), reduced viscosity (η/η_o), concentration (c) and reduced viscosity (η/η_o) at different temperatures and molality (m) for all four ILs.

m / mol·kg^{-1}	η / mPa·s	η/η_o	c / mol·dm^{-3}
**N-octylnicotinium bromide**			
T = 293.15 K			
0.02000	1.010	1.010	0.01984
0.04091	1.036	1.036	0.04034
0.05996	1.058	1.058	0.05880
0.07999	1.083	1.082	0.07797
0.09987	1.108	1.108	0.09679
T = 298.15 K			
0.02000	0.897	1.010	0.01982
0.04091	0.918	1.034	0.04029
0.05996	0.937	1.055	0.05872
0.07999	0.958	1.079	0.07787
0.09987	0.979	1.102	0.09666
T = 303.15 K			
0.02000	0.803	1.009	0.01979
0.04091	0.822	1.032	0.04023
0.05996	0.838	1.053	0.05864
0.07999	0.852	1.071	0.07776
0.09987	0.874	1.098	0.09652
T = 308.15 K			
0.02000	0.723	1.013	0.01976
0.04091	0.736	1.031	0.04017
0.05996	0.754	1.057	0.05854
0.07999	0.769	1.078	0.07762
0.09987	0.786	1.100	0.09635
T = 313.15 K			
0.02000	0.656	1.006	0.01972
0.04091	0.669	1.025	0.04009
0.05996	0.682	1.045	0.05843
0.07999	0.695	1.065	0.07748
0.09987	0.708	1.086	0.09616
**N-decylnicotinium bromide**			
T = 293.15 K			
0.00108	0.985	0.985	0.00108
0.00200	0.987	0.987	0.00200

0.00258	0.989	0.989	0.00257
0.00400	0.992	0.992	0.00399
0.00519	0.995	0.995	0.00517
T = 298.15 K			
0.00108	0.872	0.982	0.00108
0.00200	0.873	0.983	0.00199
0.00258	0.874	0.985	0.00257
0.00400	0.877	0.988	0.00398
0.00519	0.880	0.991	0.00516
T = 303.15 K			
0.00108	0.773	0.971	0.00108
0.00200	0.775	0.973	0.00199
0.00258	0.776	0.975	0.00256
0.00400	0.780	0.979	0.00398
0.00519	0.782	0.983	0.00516
T = 308.15 K			
0.00108	0.696	0.974	0.00108
0.00200	0.697	0.977	0.00199
0.00258	0.698	0.978	0.00256
0.00400	0.701	0.982	0.00397
0.00519	0.703	0.985	0.00515
T = 313.15 K			
0.00108	0.630	0.965	0.00107
0.00200	0.631	0.967	0.00198
0.00258	0.632	0.968	0.00255
0.00400	0.634	0.972	0.00396
0.00519	0.636	0.976	0.00514
**N-dodecylnicotinium bromide**			
T = 293.15 K			
0.00096	1.000	1.001	0.00096
0.00202	1.003	1.004	0.00201
0.00254	1.005	1.006	0.00253
0.00403	1.007	1.007	0.00402
0.00504	1.008	1.009	0.00502
T = 298.15 K			
0.00096	0.888	1.001	0.00096
0.00202	0.890	1.004	0.00201
0.00254	0.892	1.006	0.00253
0.00403	0.894	1.008	0.00401
0.00504	0.896	1.010	0.00502
T = 303.15 K			

0.00096	0.797	1.002	0.00096
0.00202	0.799	1.004	0.00201
0.00254	0.800	1.005	0.00252
0.00403	0.802	1.008	0.00401
0.00504	0.804	1.010	0.00501
T = 308.15 K			
0.00096	0.720	1.000	0.00096
0.00202	0.721	1.001	0.00201
0.00254	0.721	1.002	0.00252
0.00403	0.724	1.005	0.00400
0.00504	0.725	1.007	0.00500
T = 313.15 K			
0.00096	0.654	1.000	0.00095
0.00202	0.655	1.001	0.00200
0.00254	0.655	1.001	0.00252
0.00403	0.657	1.004	0.00399
0.00504	0.658	1.006	0.00499
**N-tetradecylnicotinium bromide**			
T = 293.15 K			
0.00049	0.996	0.997	0.00049
0.00078	0.997	0.997	0.00078
0.00108	0.997	0.997	0.00108
0.00199	0.998	0.999	0.00199
0.00247	0.998	0.999	0.00247
T = 298.15 K			
0.00049	0.886	0.999	0.00049
0.00078	0.886	0.999	0.00078
0.00108	0.887	0.999	0.00108
0.00199	0.887	1.000	0.00199
0.00247	0.888	1.001	0.00247
T = 303.15 K			
0.00049	0.794	0.997	0.00049
0.00078	0.794	0.998	0.00078
0.00108	0.794	0.998	0.00108
0.00199	0.795	1.000	0.00199
0.00246	0.796	1.000	0.00246
T = 308.15 K			
0.00049	0.716	0.995	0.00049
0.00077	0.717	0.995	0.00077
0.00108	0.717	0.996	0.00108
0.00198	0.717	0.996	0.00198

0.00246	0.718	0.997	0.00246
T = 313.15 K			
0.00049	0.651	0.995	0.00049
0.00077	0.651	0.995	0.00077
0.00107	0.651	0.996	0.00107
0.00198	0.652	0.997	0.00198
0.00245	0.652	0.997	0.00245

Standard uncertainties are: $u(m) = 5\cdot10^{-5}$, $u(T)$ = 0.015 K;

Relative standard uncertainty: $u_r(\eta)$ = 0.005, $u_r(p)$ = 0.015.

Table S7. The MIC and MBC values (expressed in mol·dm^{-3}) for nicotine and the four nicotine-based ionic liquids on all 12 examined microorganisms, alongside comparisons against BenCl from literature [1-5] and additional values expressed in mM and mg/L

Gr- bacteria	*E. coli*		*P. mirabills*		*P. aeruginosa*	
	MIC	MBC	MIC	MBC	MIC	MBC
Nicotine	2.50x10^{-2}	2.50x10^{-2}	2.50 x10^{-2}	2.50x10^{-2}	1.25x10^{-2}	2.50x10^{-2}
C$_8$NicBr	1.95x10^{-4}	1.95x10^{-4}	7.81x10^{-4}	7.81x10^{-4}	7.81x10^{-4}	7.81x10^{-4}
C$_{10}$NicBr	2.44x10^{-5}	4.88x10^{-5}	1.95x10^{-4}	1.95x10^{-4}	9.77x10^{-5}	3.91x10^{-4}
C$_{12}$NicBr	1.22x10^{-5}	2.44x10^{-5}	4.88x10^{-5}	9.77x10^{-5}	9.77x10^{-5}	1.95x10^{-4}
C$_{14}$NicBr	6.10x10^{-6}	2.44x10^{-5}	2.44x10^{-5}	2.44x10^{-5}	4.88x10^{-5}	4.88x10^{-5}
BenCl	2.3-4.5x10^{-5} [1]	4.5-9.0x10^{-5} [1]	/	/	0.9-1.8x10^{-4} [2]	1.8-3.6x10^{-4} [2]

Gr+ bacteria	*M. luteus*		*S. aureus*		*S. epidermidis*	
	MIC	MBC	MIC	MBC	MIC	MBC
Nicotine	1.25x10^{-2}	1.25x10^{-2}	1.25x10^{-2}	1.25x10^{-2}	1.25x10^{-2}	1.25x10^{-2}
C$_8$NicBr	2.44x10^{-5}	4.88x10^{-5}	4.88x10^{-5}	4.88x10^{-5}	9.77x10^{-5}	9.77x10^{-5}
C$_{10}$NicBr	1.53x10^{-6}	6.10x10^{-6}	6.10x10^{-6}	6.10x10^{-6}	6.10x10^{-6}	6.10x10^{-6}
C$_{12}$NicBr	6.10x10^{-6}	6.10x10^{-6}	<1.53x10^{-6}	<1.53x10^{-6}	1.22x10^{-5}	1.22x10^{-5}
C$_{14}$NicBr	<1.53x10^{-6}	<1.53x10^{-6}	<1.53x10^{-6}	<1.53x10^{-6}	<1.53x10^{-6}	<1.53x10^{-6}
BenCl	/	/	0.3-2.3x10^{-5} [3,4]	1.13-4.52x10^{-5} [3,4]	/	/

Yeasts	*C. albicans*		*C. guilliermondii*	
	MIC	MFC	MIC	MFC
Nicotine	6.25x10^{-3}	6.25x10^{-3}	6.25x10^{-3}	6.25x10^{-3}
C$_8$NicBr	1.56x10^{-3}	1.56x10^{-3}	3.91x10^{-4}	3.91x10^{-4}
C$_{10}$NicBr	9.77x10^{-5}	3.91x10^{-4}	9.77x10^{-5}	3.91x10^{-4}
C$_{12}$NicBr	4.88x10^{-5}	9.77x10^{-5}	9.77x10^{-5}	9.77x10^{-5}
C$_{14}$NicBr	4.88x10^{-5}	9.77x10^{-5}	3.91x10^{-4}	7.81x10^{-4}
BenCl	0.6-2.3x10^{-5} [2]	/	/	/

Filamentous fungi	*P. verrucosum*		*P. chrysogenum*		*A. flavus*		*A. parasiticus*	
	MIC	MFC	MIC	MFC	MIC	MFC	MIC	MFC
Nicotine	3.13x10^{-3}	3.13x10^{-3}	6.25x10^{-3}	1.25x10^{-2}	1.25x10^{-2}	ND	1.25x10^{-2}	ND
C$_8$NicBr	1.56x10^{-3}	1.56x10^{-3}	1.56x10^{-3}	1.56x10^{-3}	1.25x10^{-2}	1.25x10^{-2}	1.25x10^{-2}	1.25x10^{-2}
C$_{10}$NicBr	1.95x10^{-4}	1.95x10^{-4}	1.95x10^{-4}	1.95x10^{-4}	7.81x10^{-4}	7.81x10^{-4}	1.56x10^{-3}	3.13x10^{-3}
C$_{12}$NicBr	9.77x10^{-5}	9.77x10^{-5}	9.77x10^{-5}	9.77x10^{-5}	3.91x10^{-4}	7.81x10^{-4}	7.81x10^{-4}	7.81x10^{-4}
C$_{14}$NicBr	2.44x10^{-5}	4.88x10^{-5}	2.44x10^{-5}	2.44x10^{-5}	9.77x10^{-5}	1.95x10^{-4}	1.95x10^{-4}	1.95x10^{-4}
BenCl	/	/	/	/	2.1x10^{-7} [5]	4.2x10^{-7} [5]	4.2x10^{-7} [5]	1.27x10^{-6} [5]

Expressed in mM and mg/L

Gr- bacteria	*E. coli*				*P. mirabills*				*P. aeruginosa*			
	MIC		MBC		MIC		MBC		MIC		MBC	
	mM	mg/L	mM	mg/L	mM	mg/L	mM	mg/L	mM	mg/L	mM	mg/L
Nicotine	25.0	8111	25.0	8111	25.0	8111	25.0	8111	12.5	4056	25.0	8111
C$_8$NicBr	1.95x10^{-1}	69.4	1.95x10^{-1}	69.4	7.81x10^{-1}	278	7.81x10^{-1}	278	7.81x10^{-1}	278	7.81x10^{-1}	278
C$_{10}$NicBr	2.44x10^{-2}	9.36	4.88x10^{-2}	18.7	1.95x10^{-1}	74.9	1.95x10^{-1}	74.9	9.77x10^{-2}	37.4	3.91x10^{-1}	300
C$_{12}$NicBr	1.22x10^{-2}	5.02	2.44x10^{-2}	10.1	4.88x10^{-2}	20.1	9.77x10^{-2}	40.2	9.77x10^{-2}	40.2	1.95x10^{-1}	80.4
C$_{14}$NicBr	6.10x10^{-3}	2.68	2.44x10^{-2}	10.7	2.44x10^{-2}	10.7	2.44x10^{-2}	10.7	4.88x10^{-2}	21.5	4.88x10^{-2}	21.5

Gr+ bacteria	*M. luteus*				*S. aureus*				*S. epidermidis*			
	MIC		MBC		MIC		MBC		MIC		MBC	
	mM	mg/L	mM	mg/L	mM	mg/L	mM	mg/L	mM	mg/L	mM	mg/L
Nicotine	12.5	4056	12.5	4056	12.5	4056	12.5	4056	12.5	4056	12.5	4056
C$_8$NicBr	2.44x10^{-2}	8.68	4.88x10^{-2}	17.4	4.88x10^{-2}	17.4	4.88x10^{-2}	17.4	9.77x10^{-2}	34.7	9.77x10^{-2}	34.7
C$_{10}$NicBr	1.53x10^{-3}	0.59	6.10x10^{-3}	2.34	6.10x10^{-3}	2.34	6.10x10^{-3}	2.34	6.10x10^{-3}	2.34	6.10x10^{-3}	2.34
C$_{12}$NicBr	6.10x10^{-3}	2.51	6.10x10^{-3}	2.51	<1.53x10^{-3}	<0.63	<1.53x10^{-3}	<0.63	1.22x10^{-2}	5.02	1.22x10^{-2}	5.02
C$_{14}$NicBr	<1.53x10^{-3}	<0.67	<1.53x10^{-3}	<0.67	<1.53x10^{-3}	<0.67	<1.53x10^{-3}	<0.67	<1.53x10^{-3}	<0.67	<1.53x10^{-3}	<0.67

Yeasts	*C. albicans*				*C. guilliermondii*			
	MIC		MFC		MIC		MFC	
	mM	mg/L	mM	mg/L	mM	mg/L	mM	mg/L
Nicotine	6.25	1014	6.25	1014	6.25	1014	6.25	1014

C_8NicBr	1.56	554.4	1.56	554.4	3.91×10^{-1}	138.8	3.91×10^{-1}	138.8
C_{10}NicBr	9.77×10^{-2}	37.4	3.91×10^{-1}	149.8	9.77×10^{-2}	37.4	3.91×10^{-1}	149.8
C_{12}NicBr	4.88×10^{-2}	20.1	9.77×10^{-2}	40.2	9.77×10^{-2}	40.2	9.77×10^{-2}	40.2
C_{14}NicBr	4.88×10^{-2}	10.7	9.77×10^{-2}	10.7	3.91×10^{-1}	171.7	7.81×10^{-1}	343.4
Filamentous fungi	*P. verrucosum*				*P. chrysogenum*			
	MIC		MFC		MIC		MFC	
	mM	mg/L	mM	mg/L	mM	mg/L	mM	mg/L
Nicotine	3.13	508	3.13	508	6.25	1014	12.5	2028
C_8NicBr	1.56	554	1.56	554	1.56	554	1.56	554
C_{10}NicBr	1.95×10^{-1}	74.9	1.95×10^{-1}	74.9	1.95×10^{-1}	74.9	1.95×10^{-1}	74.9
C_{12}NicBr	9.77×10^{-2}	40.2	9.77×10^{-2}	40.2	9.77×10^{-2}	40.2	9.77×10^{-2}	40.2
C_{14}NicBr	2.44×10^{-2}	10.7	4.88×10^{-2}	21.4	2.44×10^{-2}	10.7	2.44×10^{-2}	10.7
Filamentous fungi	*A. flavus*				*A. parasiticus*			
	MIC		MFC		MIC		MFC	
	mM	mg/L	mM	mg/L	mM	mg/L	mM	mg/L
Nicotine	12.5	2028	ND	ND	12.5	2028	ND	ND
C_8NicBr	12.5	8884	12.5	8884	12.5	8884	12.5	8884
C_{10}NicBr	7.81×10^{-1}	299	7.81×10^{-1}	299	1.56	598	3.13	1200
C_{12}NicBr	3.91×10^{-1}	161	7.81×10^{-1}	321	7.81×10^{-1}	321	7.81×10^{-1}	321
C_{14}NicBr	9.77×10^{-2}	42.9	1.95×10^{-1}	85.8	1.95×10^{-1}	85.8	1.95×10^{-1}	85.8

*<1.53×10^{-6} - MIC/MBC below minimum examined concentration

**ND - not detected, above maximum tested concentration

[1] M. Ligowska-Marzeta, V. Hancock, H. Ingmer, F.M. Aerestrup, Comparison of Gene Expression Profiles of Uropathogenic *Escherichia Coli* CFT073 after Prolonged Exposure to Subinhibitory Concentrations of Different Biocides. Antibiotics. 8(4) (2019) 164. https://doi.org/10.3390/antibiotics8040167

[2] M. Stupar, M. Ljaljević Grbić, G. Subakov Simić, A. Jelikić, J. Vukojević, M. Sabovljević. A sub-aerial biofilms investigation and new approach in biocide application in cultural heritage conservation: Holy Virgin Church (Gradac Monastery, Serbia). Indoor and Built Environ. 23(4) (2014) 584-593. https://doi.org/10.1177/1420326X12466753

[3] E.C.R. Bonsaglia, G.H. Calvo, D.O. Sordelli, N.C.C. Silva, V.L.M. Rall, A. Casas, F. Buzzola, The Impact of Low-Level Benzalkonium Chloride Exposure on *Staphylococcus* spp. Strains and Control by Photoinactivation. Antibiotics. 12(8) 2023 1244. https://doi.org/10.3390/antibiotics12081244

[4] G.H. Lee, S.H. Park, B.M. Song, D.M. Kim, H.J. Han, J.Y. Park, Y.W. Jo, M.Y. Hwang, K.T. Sim, S.M. Kang, D. Tark, Comparative efficacy evaluation of disinfectants against severe acute respiratory syndrome coronavirus-2. J. Hosp. Infect. 131 (2022) 12-22. https://doi.org/10.3390/antibiotics12081244

[5] N. Unković, M. Ljaljević Grbić, M. Stupar, J. Vukojević, V. Janković, D. Jović, A. Djordjević, Aspergilli Response to Benzalkonium Chloride and Novel-Synthesized Fullerenol/Benzalkonium Chloride Nanocomposite. Sci. Word. J. 1 (2015) 109262. https://doi.org/10.1155/2015/109262

Table S8. ^{1}H and ^{13}C NMR spectra assignments for all four ionic liquids

N-octylnicotinium bromide

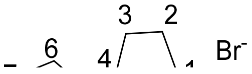

Solvent: D_2O

T = 298.15 K

^{1}H NMR assignments				
Hi	δ(^{1}H) / ppm	multeplicity	J / Hz	integrals
H9	8.82	s		2
H8	8.81	d	∽6,0	
H6	8.53	d	8.1	1
H7	8.10	dd	7,9; 6,4	1
H11	4.63	t	7.2	2
H4	3.59	t	8.5	1
H1a	3.24	m		1
H1b	2.52	q	8.3	1
H3a	2.43	m		1
H10	2.23	s		3
H2,H12	2.00	m		4
H3b	1.88	m		1
H13-H17	1.29	m		10
H18	0.85	pseudot	∽6,0	3

^{13}C NMR assignments		
Ci	δ(^{13}C) / ppm	HSQC correlations
C6	144.5	H6
C5	143.4	-
C8, C9	143.3	H8,H9
C7	128.3	H7
C4	67.4	H4
C11	62.1	H11
C1	56.4	H1
C10	39.3	H10
C3	33.6	H3
C13-C16 (1C)	30.9	H13-H17
C12	30.4	H12
	28.1	
C13-C16 (3C)	28.0	H13-H17
	25.1	
C2	22.1	H2
C17	22.0	H17
C18	13.4	H18

N-decylnicotinium bromide

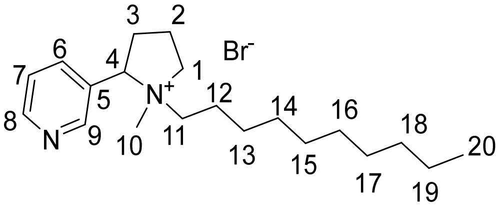

Solvent: D_2O

T = 298.15 K

^{1}H NMR assignments				
Hi	δ(^{1}H) / ppm	multeplicity	J / Hz	integrals
H8	9.00	d	∽6,0	2
H9	8.99	s		

H6	8.62	d	8.1	1
H7	8.19	pseudot	7.2	1
H11	4.77	t	7.0	2
H4	3.65	t	8.5	1
H1a	3.25	m		1
H1b	2.51	q	9.0	2
H3a	2.43	m		1
H10	2.25	s		3
H12	2.07	m		4
H2	1.98	m		1
H3b	1.86	m		1
H13-H19	1.23	m		14
H20	0.75	pseudot	∽6,5	3

^{13}C NMR assignments

Ci	δ(^{13}C) / ppm	HSQC correlations
C6	144.7	H6
C5	143.8	-
C8, C9	143.6<br>143.3	H8,H9
C7	128.6	H7
C4	67.3	H4
C11	61.8	H11
C1	56.5	H1
C10	39.5	H10
C3	34.0	H3
C13-C19 (1C)	31.6	H13-H19
C12	31.0	H12
C13-C19 (6C)	29.2<br>29.1<br>29.0<br>28.7<br>25.6<br>22.4	H13-H19
C2	22.3	H2
C20	13.7	H20

N-dodecylnicotinium bromide

Solvent: D_2O

T = 298.15 K

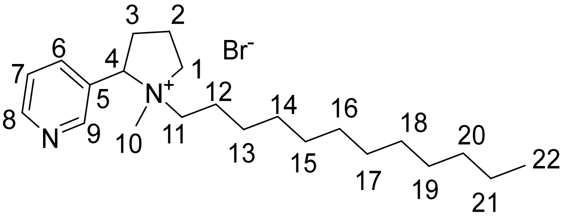

^{1}H NMR assignments

Hi	δ(^{1}H) / ppm	multeplicity	J / Hz	integrals
H8	9.06	d	∽6,0	2
H9	9.04	s		
H6	8.64	d	8.0	1
H7	8.21	dd	7,8; 6,5	1
H11	4.82	t	7.0	-

H4	3.66	t	8.1	1
H1a	3.25	m		1
H1b	2.49	q	9.0	2
H3a	2.43	m		1
H10	2.25	s		3
H12	2.09	m		5
H2	1.97	m		1
H3b	1.85	m		10
H13-H21	1.23	m		18
H22	0.75	pseudot	∽6,5	3

^{13}C NMR assignments

Ci	δ(^{13}C) / ppm	HSQC correlations
C6	144.8	H6
C5	144.0	-
C8, C9	143.6<br>143.3	H8,H9
C7	128.7	H7
C4	67.3	H4
C11	61.8	H11
C1	56.6	H1
C10	39.6	H10
C3	34.2	H3
C13-C21 (1C)	31.8	H13-H21
C12	31.2	H12
C13-C21 (8C)	29.7<br>29.65<br>29.63<br>29.5<br>29.3<br>29.0<br>25.8<br>22.5	H13-H21
C2	22.4	H2
C22	13.8	H22

N-tetradecylnicotinium bromide

Solvent: D_2O

T = 298.15 K

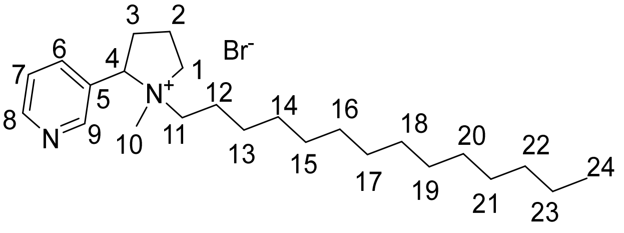

^{1}H NMR assignments

Hi	δ(^{1}H) / ppm	multeplicity	J / Hz	integrals
H8	9.09	d	6.0	1
H9	9.06	s		1
H6	8.65	d	8.4	1
H7	8.22	dd	7,9; 6,2	1
H11	4.84	t	7.0	2

H4	3.66	t	8.2	1
H1a	3.24	m		1
H1b	2.49	q	9.0	2
H3a	2.43	m		1
H10	2.25	s		3
H12	2.10	m		5
H2	1.97	m		1
H3b	1.85	m		10
H13-H23	1.25	m		22
H24	0.78	pseudot	∽6,6	3

^{13}C NMR assignments

Ci	δ(^{13}C) / ppm	HSQC correlations
C6	144.8	H6
C5	144.2	-
C8, C9	143.6	H8,H9
	143.3	
C7	128.7	H7
C4	67.3	H4
C11	61.7	H11
C1	56.6	H1
C10	39.7	H10
C3	34.3	H3
C13-C23 (1C)	31.9	H13-H23
C12	31.3	H12
	29.89	
	29.88	
	29.79	
	29.77	
C13-C23 (10C)	29.6	H13-H23
	29.4	
	29.1	
	25.9	
	22.6	
C2	22.4	H2
C22	13.8	H22

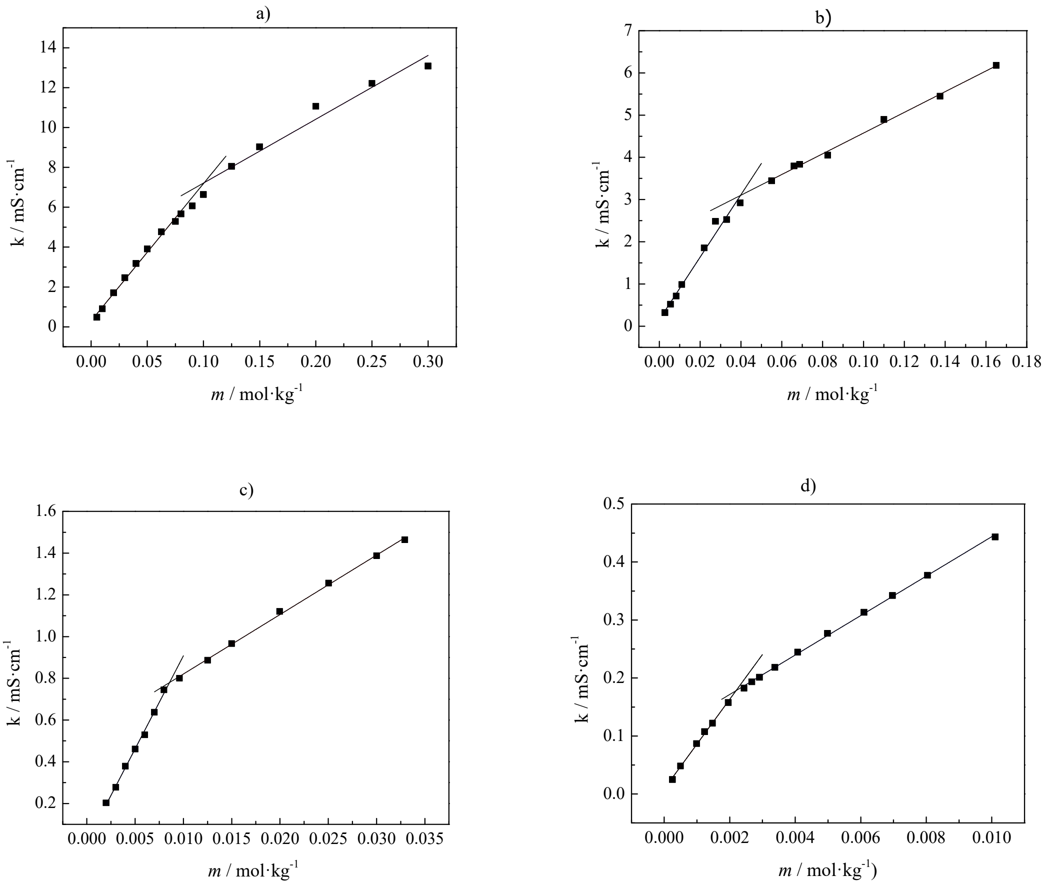

Figure S1. Dependence of conductivity *k* versus molality *m* of aqueous solutions of: a) C_8NicBr, b) C_{10}NicBr c) C_{12}NicBr, d) C_{14}NicBr

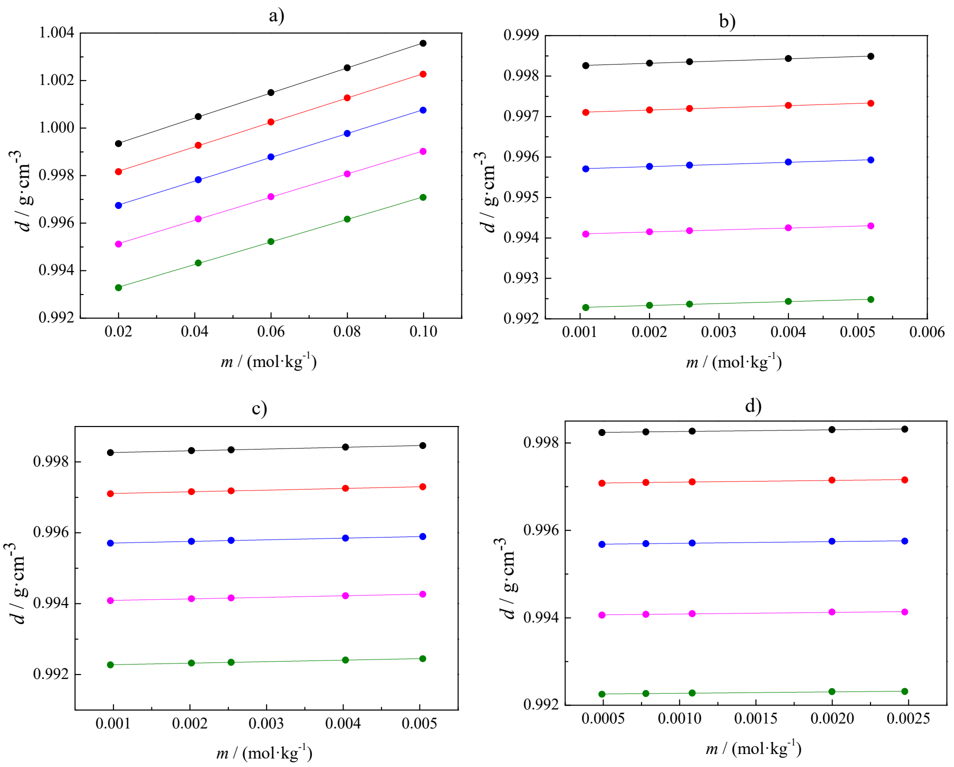

Figure S2. Variation of density of aqueous solutions of: a) C_8NicBr ; b) C_{10}NicBr c) C_{12}NicBr d) C_{14}NicBr with molality at different temperatures: ● 293.15; ● 298.15; ● 303.15; ● 308.15; ● 313.15 K.

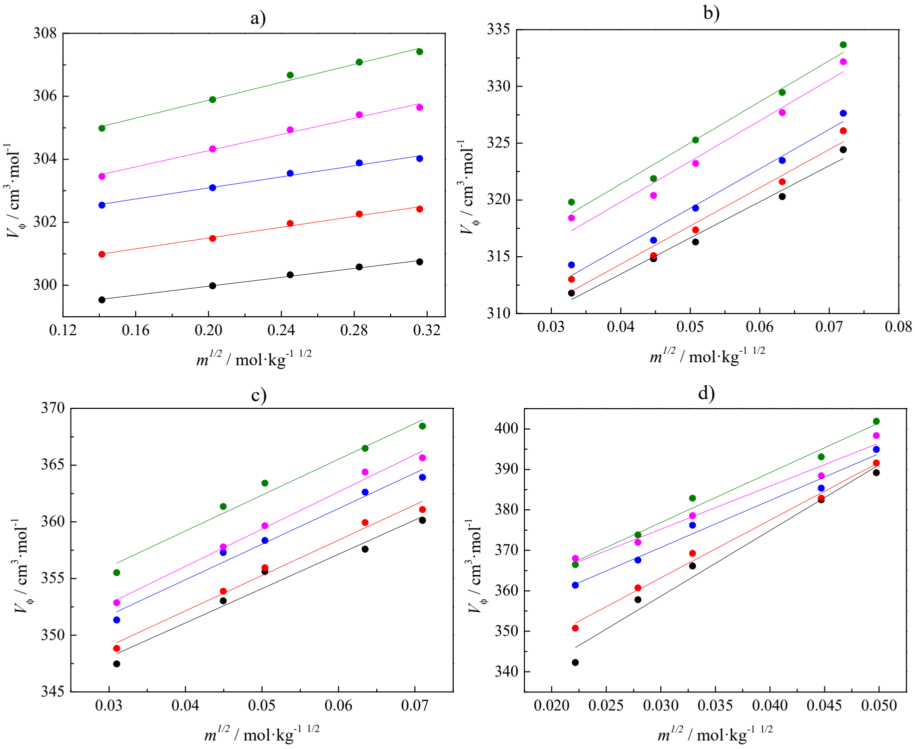

Figure S3. Linear fit of the square of molality of: a a) C_8NicBr ; b) C_{10}NicBr c) C_{12}NicBr d) C_{14}NicBr with apparent molar volume (V_ϕ) as per *Masson*'s equation, at different temperatures: ● 293.15; ● 298.15; ● 303.15; ● 308.15; ● 313.15 K.

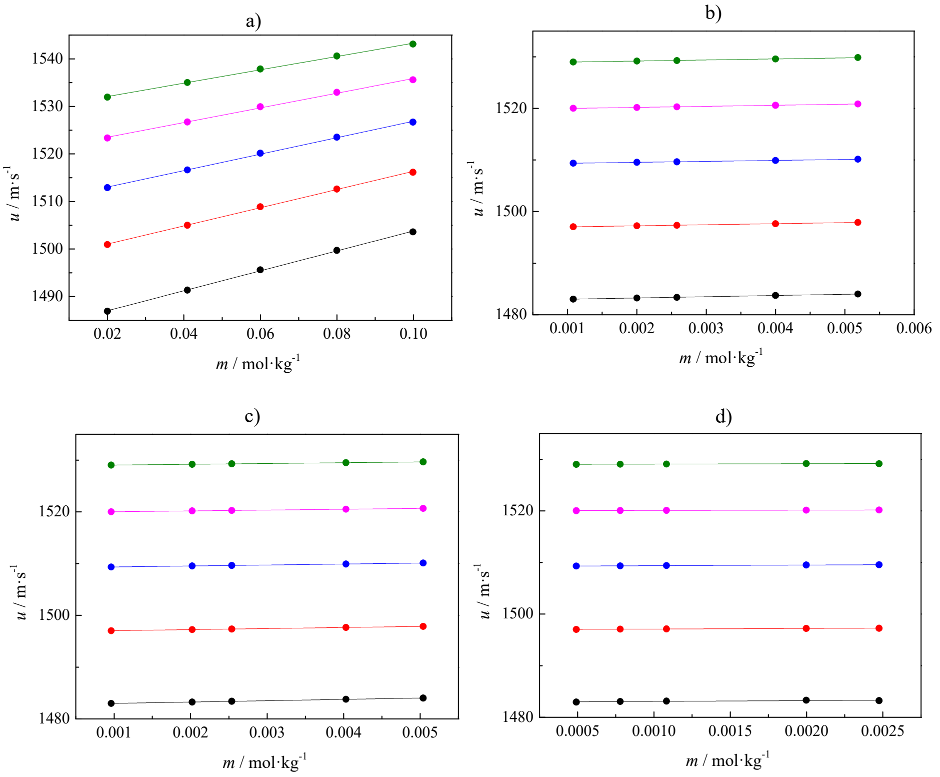

Figure S4. Variation of speed of sound in aqueous solutions of: a) C_8NicBr ; b) C_{10}NicBr c) C_{12}NicBr d) C_{14}NicBr with molality at different temperatures: ● 293.15; ● 298.15; ● 303.15; ● 308.15; ● 313.15 K.

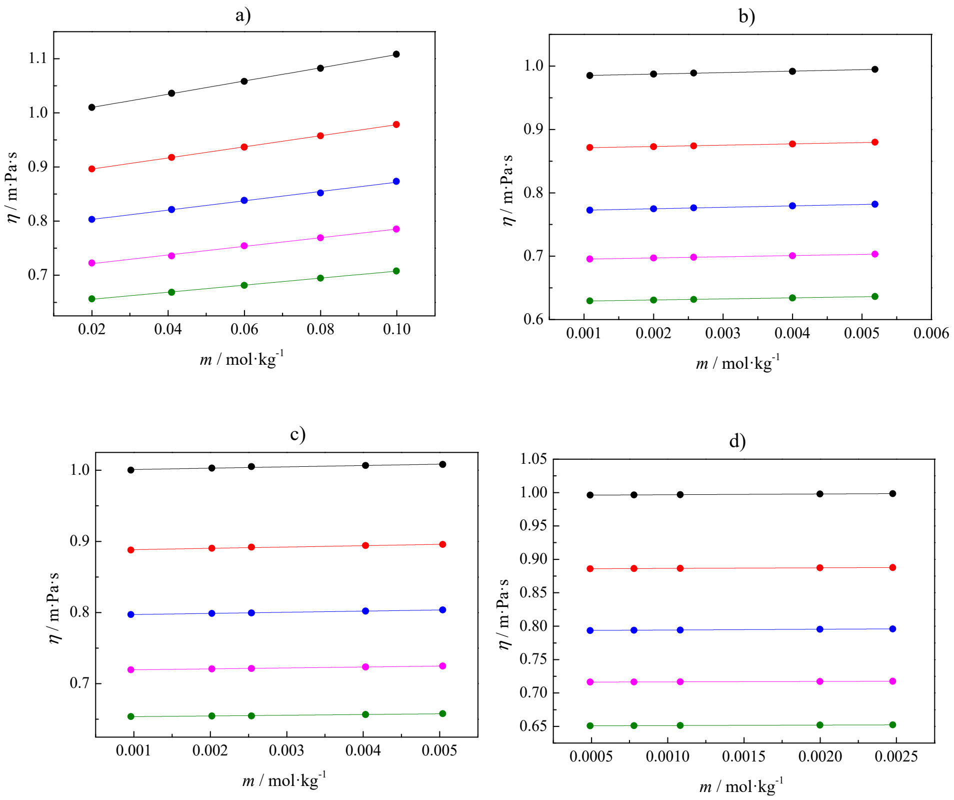

Figure S5. Variation of viscosity in aqueous solutions of: a) C_8NicBr ; b) C_{10}NicBr c) C_{12}NicBr d) C_{14}NicBr with molality at different temperatures: ● 293.15; ● 298.15; ● 303.15; ● 308.15; ● 313.15 K.

a)

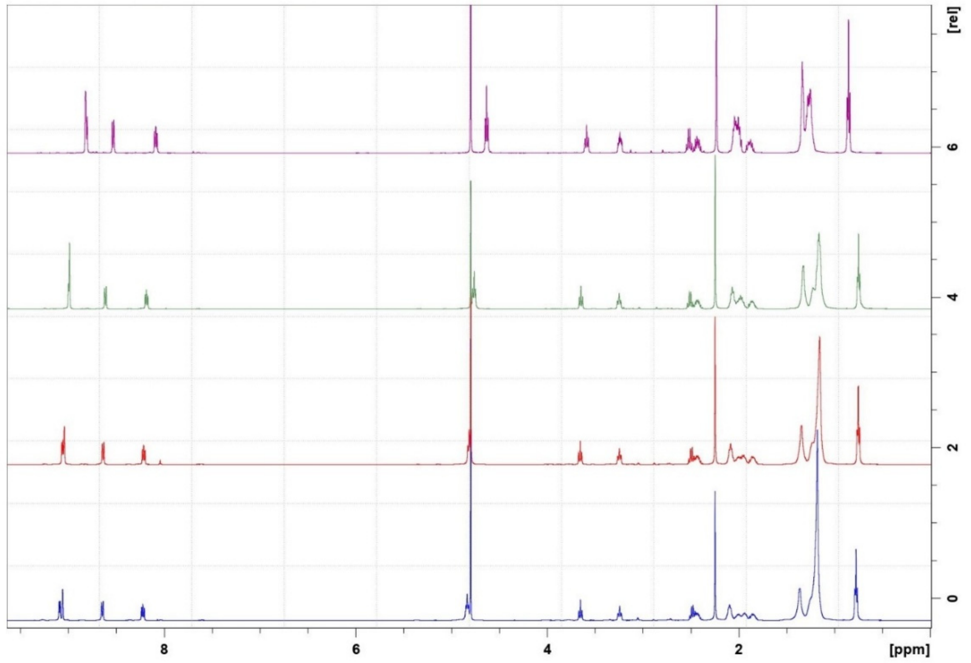

b)

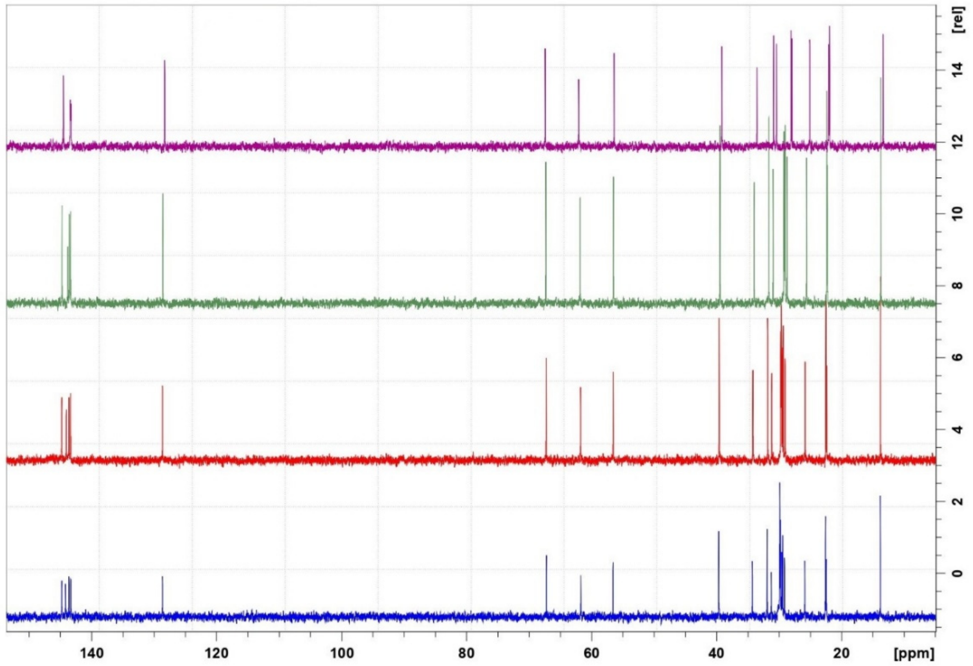

Figure S6. a) ^{1}H and b) ^{13}C NMR spectrum for ■ N-octylnicotinium bromide ■ N-decylnicotinium bromide; ■ N-dodecylnicotinium bromide; ■ N-tetradecylnicotinium bromide
